# Ambient RF energy harvesting using voltage doubler rectifier for battery-free agricultural sensor networks: Design, statistical analysis, and machine learning validation

**DOI:** 10.1371/journal.pone.0350236

**Published:** 2026-09-10

**Authors:** Md. Atik Hasan Nishat, Prithwiraj Biswas Pallab, Nowrin Jannat, Saleha Nasrin Mishu, Md Fahad Ullah Utsho, Md. Bipul Islam, Riaz Uddin Mondal, Md. Firoz Ahmed, M. Hasnat Kabir

**Affiliations:** Department of Information and Communication Engineering, University of Rajshahi, Rajshahi, Bangladesh; Galgotias College of Engineering and Technology, Greater Noida, INDIA

## Abstract

The growing adoption of precision agriculture requires sustainable power sources for wireless sensor networks (WSNs) deployed in remote and resource-constrained environments. Conventional battery-powered systems are constrained by limited-service life, maintenance requirements, and environmental concerns. This study presents the design, experimental validation, statistical evaluation, and machine learning (ML)-based modeling of an ambient radio-frequency (RF) energy harvesting system for agricultural monitoring applications. The proposed system harvests RF energy from AM/FM broadcasting (558 kHz–108 MHz) and cellular communication bands (800–2100 MHz) using a frequency-selective broadband antenna, an integrated diplexer, switchable L-section impedance-matching networks, and a Villard voltage-doubler rectifier based on low forward-voltage OA79 germanium diodes. Experimental measurements from 30 independent repeated trials yielded a mean open-circuit output voltage of 4.07 ± 0.08 V (95% CI: 4.04–4.10 V) and a loaded output voltage of 2.11 ± 0.11 V, corresponding to a harvested power of approximately 44.5 μW across a 100 kΩ load. One-way analysis of variance (ANOVA) demonstrated significant differences among diode types (F (2,87) = 687.4, p < 0.001), while Tukey’s post hoc test confirmed the superior performance of OA79 diodes compared with 1N4148 and 1N5819 alternatives. Among the evaluated ML models, Gradient Boosting regression achieved the highest predictive accuracy, with R² = 0.963 and RMSE = 0.071 V. SHapley Additive exPlanations (SHAP) analysis identified diode forward voltage and ambient RF power density as the most influential factors affecting output voltage prediction. The results indicate the potential applicability of ambient multi-band RF energy harvesting as a supplementary energy source for low-power agricultural sensing applications operating under duty-cycled conditions. By combining broadband energy harvesting, statistical validation, and predictive modeling, the proposed framework establishes a systematic methodology for evaluating self-powered agricultural IoT systems operating under variable RF environments.

## 1. Introduction

Smart agriculture and precision farming increasingly rely on wireless sensor networks (WSNs) for real-time monitoring of environmental and crop-related parameters, including soil moisture, temperature, and humidity. These systems enable data-driven irrigation, fertilizer management, and pest control, improving resource efficiency and crop productivity [1,2]. However, long-term deployment of agricultural WSNs remains significantly constrained by dependence on conventional battery-based power supplies. In large-scale and remote environments, battery replacement is often labor-intensive and costly, contributing significantly to operational expenses [3]. In addition, improper disposal of batteries introduces environmental risks due to hazardous materials such as lead, lithium, and cadmium [3].

RF energy harvesting has been extensively investigated as a potential supplementary power source for ultra-low-power electronic and wireless sensing systems [4]. It enables conversion of ambient electromagnetic energy into usable electrical power from existing RF infrastructure, including cellular base stations (800–2100 MHz), FM broadcast transmitters (88–108 MHz), and AM radio services (558–1089 kHz). Unlike solar or wind energy harvesting, ambient RF energy may remain available independent of daylight conditions and is generally less sensitive to short-term environmental variations [4]. Reported ambient RF power densities vary considerably with transmitter density, propagation conditions, and measurement location, typically ranging from approximately 0.1–10 µW/cm² in rural environments and reaching several tens of microwatts per square centimeter in semi-urban areas located closer to RF transmitters [5–8]. Although these levels are relatively low compared to other renewable sources, they can still support ultra-low-power sensing systems operating in duty-cycled modes with average power consumption below 100 µW [9].

Despite significant research progress, several technical limitations remain in existing RF energy harvesting studies. First, many reported systems are evaluated using controlled signal sources or simplified laboratory setups that do not fully capture the variability of real ambient RF environments, where multiple transmitters, fading, and temporal fluctuations coexist [10]. Second, performance evaluation of rectifier components is often based on limited experimental trials, with relatively few studies incorporating formal statistical significance testing. Third, Third, the application of machine learning techniques for modeling and interpreting RF energy harvesting performance remains relatively limited. Finally, most existing designs focus on single-band or narrowband operation, restricting applicability in heterogeneous RF environments [11,12].

To address these limitations, this work develops and experimentally evaluates a multi-band RF energy harvesting system operating across AM, FM, and cellular communication frequency bands for agricultural IoT applications. Unlike many prior studies that rely on single-tone or fixed-power excitation, the experimental platform in this work is designed to emulate realistic ambient RF variability using multi-frequency excitation combined with controlled time-varying power levels. This approach enables repeatable laboratory experimentation while approximating key statistical characteristics of real-world RF fluctuations, which are difficult to capture consistently in outdoor measurements due to environmental variability and infrastructure constraints. From a methodological perspective, the proposed system integrates band-specific impedance matching networks, statistical evaluation, and machine learning-based modeling. Rectifier diode performance is assessed using Analysis of Variance (ANOVA) and Tukey’s Honest Significant Difference (HSD) post-hoc testing to ensure statistically meaningful comparisons across repeated measurements. In addition, machine learning models are employed to predict harvested output voltage under varying operating conditions, while SHAP (SHapley Additive exPlanations) analysis is used to quantify the contribution of key system parameters to system performance. From an application perspective, the proposed architecture is intended for self-powered agricultural sensing platforms, including soil moisture monitoring, nutrient sensing, environmental tracking nodes, and low-duty-cycle telemetry devices. These applications are particularly relevant in rural and resource-constrained regions where grid access is limited and battery maintenance is impractical. By integrating multi-band RF harvesting, statistical performance validation, and interpretable machine-learning-based prediction within a unified framework, this study provides a practical methodology for evaluating ambient RF energy harvesting systems for precision agriculture applications.

The main contributions of this work are as follows:

1. Design and experimental evaluation of a multi-band RF energy harvesting system covering AM, FM, and cellular frequency bands relevant to agricultural IoT environments.
2. Development of band-specific impedance matching networks to improve power transfer efficiency across widely separated frequency bands.
3. Statistical evaluation of rectifier diode performance using ANOVA, confidence intervals, and Tukey HSD post-hoc testing for robust comparison under repeated measurements.
4. Integration of machine-learning models for harvested voltage prediction together with SHAP-based interpretability analysis to examine the influence of operational parameters on system performance.
5. Experimental validation conducted under controlled laboratory conditions was designed to emulate key statistical characteristics of ambient RF variability, enabling repeatable and comparative evaluation while establishing a foundation for future field deployment studies.

## 2. Methodology

### 2.1. System architecture overview

**Fig 1:**
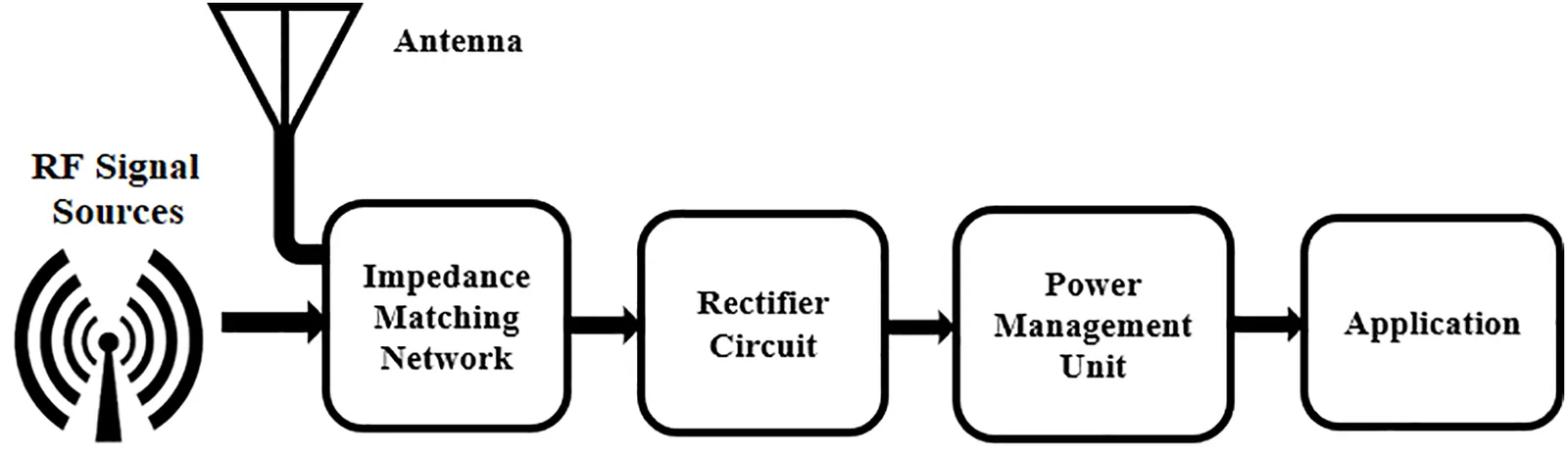
Block diagram of the proposed RF energy harvesting system.

The proposed RF energy harvesting system converts ambient electromagnetic energy into usable DC power through a four-stage architecture: (i) RF energy capture via a broadband frequency-selective antenna; (ii) impedance matching network for maximum power transfer; (iii) RF-to-DC rectification with voltage multiplication; and (iv) energy storage and output filtering. The system is designed to target ambient RF sources commonly present in rural and semi-urban agricultural regions of South Asia: AM Band (558–1089 kHz), FM Band (88–108 MHz), and Cellular Bands (800–2100 MHz) – consistent with previously reported RF infrastructure availability studies conducted in this region [4]. Fig 1 illustrates the block diagram of the proposed RF energy harvesting system, depicting the complete signal flow from RF energy capture through impedance matching, rectification, and energy storage stages. The architecture demonstrates the conversion pathway of ambient radio-frequency signals into usable DC power for supplying low-power agricultural sensor nodes.

#### 2.1.1. Antenna and impedance matching network.

A frequency-selective multi-antenna system was developed to harvest ambient RF energy over the 558 kHz–2.1 GHz frequency range. Because maintaining stable gain and acceptable impedance characteristics across such a broad spectrum using a single antenna is technically challenging, three dedicated antennas were employed, each optimized for a specific frequency band. A ferrite-rod antenna (≈ −15 dBi) was used for AM broadcast reception, a half-wave dipole antenna (approximately 2.1 dBi) was designed for the FM band (98 MHz), and a planar monopole antenna with a gain of 1.8–3.2 dBi was implemented for the cellular bands (800–2100 MHz). A passive diplexer directed the received signals to the appropriate frequency branch while maintaining electrical isolation between the operating bands. To compensate for frequency-dependent impedance variations, separate L-section impedance-matching networks were designed for the AM (558 kHz–1.7 MHz), FM (88–108 MHz), and cellular (800–2100 MHz) bands instead of using a single broadband matching network. The AM matching network consisted of a 220 µH ferrite-core inductor and a 470 pF trimmer capacitor tuned near 639 kHz. For the FM band, impedance matching was achieved using a 68 nH inductor and a 1–10 pF variable capacitor optimized at 98 MHz. The cellular matching network was implemented on an FR-4 substrate ($\varepsilon_{r}$ = 4.3, thickness = 1.6 mm) using a 50 Ω tapered microstrip transformer together with tunable shunt inductors (2.2–15 nH) and series capacitors (0.5–3 pF), providing impedance matching over the 800–2100 MHz frequency range, including the lower, middle, and upper cellular sub-bands. The three matching branches were connected through an RF switch (Mini-Circuits ZSWA-2–50DR) controlled by a low-power microcontroller that selected the appropriate frequency band according to the received signal strength. During experimental characterization, the RF switch and microcontroller were powered by an external regulated bench power supply rather than by the harvested RF energy. Consequently, the reported open-circuit voltage, loaded output voltage, harvested power (approximately 44.5 µW), and RF-to-DC conversion efficiency (approximately 9%) represent only the performance of the passive RF energy harvesting chain, comprising the antenna, impedance-matching network, and voltage-doubler rectifier, and therefore exclude the power consumption of the control electronics. This distinction is important because, in a fully autonomous battery-free implementation, the control circuitry would also need to operate from the harvested energy or be replaced by a passive or duty-cycled band-selection mechanism. In such a configuration, part of the harvested energy would be consumed by the control circuitry, reducing the net power available for the sensing system. Employing an externally powered control circuit during laboratory characterization enabled independent optimization and evaluation of the impedance-matching networks without introducing additional power consumption into the measured RF-to-DC conversion performance. Experimental measurements yielded a voltage standing wave ratio (VSWR) below 2 across all targeted frequency bands, specifically below 1.8 for the AM band, below 1.6 for the FM band, and below 1.9 for the cellular bands, indicating satisfactory impedance matching under the investigated operating conditions. A detailed summary of the antenna and impedance-matching performance for each frequency band is presented in Table 1. Overall, the proposed architecture employs a switched multi-band impedance-matching strategy rather than a single broadband matching network, thereby reducing impedance mismatch and minimizing performance degradation caused by the large reactance variations associated with ultra-wideband operation.

**Table 1:** Antenna and matching network performance across sub-bands.

Sub-Band	Frequency Range	Antenna Type	Gain (dBi)	VSWR	Matching Network
AM	558 kHz-1.7 MHz	Ferrite rod	–15 to –12	< 1.8	220 µH + 470 pF (L-section)
FM	88-108 MHz	Half-wave dipole	1.8–2.3	< 1.6	68 nH + 1–10 pF (L-section)
Cellular	800-2100 MHz	Planar monopole	1.8–3.2	< 1.9	Microstrip transformer

### 2.2. RF-to-DC rectification and voltage multiplication

A Villard voltage doubler topology was selected in preference to several conventional rectifier configurations, including half-wave, full-wave bridge, and Dickson charge pump circuits, due to its inherent 2 × voltage gain, low component count (two diodes and two capacitors), and suitability for low-amplitude RF inputs. Under low RF input power conditions, rectification efficiency is strongly influenced by diode forward-voltage ($V_{f}$) characteristics. OA79 germanium diodes $\left(V_{f}\;\approx \;\text{0}.\text{2}-\text{0}.\text{3}\;V\right)$ were selected as the primary rectifier implementation, with comparative testing against 1N5819 Schottky $\left(V_{f}\approx \;\text{0}.\text{3}-\text{0}.\text{4}\;V\right)$ and 1N4148 silicon $(V_{f}\;\approx \;\text{0}.\text{7}\;V)\;$diodes. RF-to-DC conversion efficiency (η) was calculated as:


$$\begin{array}{c} \eta =\;\frac{P_{out,\;DC}}{P_{in,\;RF}}\times \text{1}\text{0}\text{0}\% \end{array}$$ (1)

where $P_{out,\;DC}=\frac{V_{load}^{\text{2}}}{R_{load}}$ is the incident RF power measured at the antenna terminals (using a calibrated spectrum analyzer and known antenna factor). Under typical ambient conditions (RF power density ≈ 0.5 µW/cm², effective antenna aperture ≈ 10 cm²), the harvested DC power was approximately 44.5 µW into a 100 kΩ load, corresponding to an estimated RF-to-DC conversion efficiency of approximately 9% for the OA79-based rectifier. The RF power density range considered in this study (0.1–5 µW/cm²) was selected based on representative values reported in previous measurement studies conducted in comparable rural and semi-urban RF environments of South Asia [5–8]. A summary of the key design parameters and component specifications is provided in Table 2.

**Table 2:** Key design parameters and component specifications.

Parameter	Value	Justification
**Diode**	OA79 (Germanium)	$V_{f}\;=\;\text{0}.\text{2}-\text{0}.\text{3}\;V$ (lowest rectification loss under low RF input)
**Rectifier Topology**	Voltage Doubler	2 × voltage gain, minimal component count, reduced cumulative loss
**Storage Capacitors**	47 µF × 2	Balances energy storage, size constraints, and output ripple suppression
**Coupling Capacitor**	0.1 µF	DC blocking; maintains efficient AC coupling across targeted RF range
**Target Frequency**	558 kHz–2.1 GHz	Covers AM/FM broadcast and GSM-900/1800, UMTS, LTE cellular bands
**Expected Output (Loaded)**	>2 V	Sufficient for ultra-low-power sensors in duty-cycled operation
**RF-to-DC Efficiency (measured)**	≈9% (at 0.5 µW/cm²)	Typical for passive rectifiers under low ambient power

### 2.3. Statistical validation framework

To improve the reliability and reproducibility of the experimental findings, all performance metrics were evaluated using 30 independent measurement trials (n = 30) for each operating condition. A comprehensive statistical validation framework was employed to quantify measurement variability, verify analytical assumptions, and assess the significance of observed performance differences among rectifier configurations.

- **Descriptive Statistical Analysis:** Descriptive statistics, including the mean, standard deviation (SD), minimum value, maximum value, coefficient of variation (CV), and 95% confidence interval (CI), were calculated for all measured output-voltage datasets. These statistical metrics provide a quantitative assessment of central tendency, data dispersion, and measurement consistency.
- **Normality Assessment:** Prior to inferential analysis, the distribution of each dataset was examined using the Shapiro–Wilk normality test, which is recommended for sample sizes smaller than 50. All experimental datasets satisfied the normality assumption (*p* > 0.05), thereby supporting the use of parametric statistical methods.
- **Analysis of Variance (ANOVA):** A one-way analysis of variance (ANOVA) was conducted to assess whether statistically significant differences exist in the mean harvested output voltages among the three rectifier diode configurations (OA79, 1N4148, and 1N5819). Statistical significance was assessed using a conventional threshold of α = 0.05.
- **Tukey Honest Significant Difference (HSD) Post-Hoc Analysis:** Following a significant ANOVA result, the Tukey Honest Significant Difference (HSD) test was performed to identify specific pairwise differences between diode configurations while controlling the family-wise error rate associated with multiple comparisons.
- **Pearson Correlation Analysis:** Pearson’s correlation coefficient (r) was computed to assess the strength and direction of the linear relationship between ambient RF power density and harvested DC output voltage, providing insight into how variations in available RF energy influence system performance.
- **Statistical Software and Significance Criteria:** All statistical analyses were performed using standard scientific computing tools, and all inferential results were considered statistically significant when p < 0.05.

### 2.4. Machine learning validation framework

A supervised machine learning framework was developed to predict harvested DC output voltage from measurable operational parameters and to quantify feature importance. The experimental dataset comprised 300 observations collected across varying RF input conditions, diode types, load resistances, and ambient temperatures. Input features: (i) diode forward voltage $(V_{f},\;V);$ (ii) input RF power density (µW/cm²); (iii) load resistance (kΩ); (iv) operating frequency band (categorical: AM/FM/Cellular); (v) ambient temperature (°C). Target variable: loaded DC output voltage (V). Six supervised regression algorithms with different model complexities were comparatively evaluated: Linear Regression, Ridge Regression (λ=0.1), Support Vector Regression (RBF kernel), Random Forest (100 estimators), Gradient Boosting (XGBoost variant, 200 estimators, learning rate 0.05), and a simple feedforward neural network (replacing LSTM, as the dataset size (300 samples) is insufficient for time-series LSTM without risk of overfitting). All models were trained using 80% of the dataset and evaluated using a held-out 20% test set, with 5-fold cross-validation to ensure generalizability. Evaluation metrics: R², RMSE, MAE, MAPE, and cross-validated RMSE. SHAP (SHapley Additive exPlanations) analysis was applied to the best-performing Gradient Boosting model to enhance interpretability and quantify the contribution of individual features to model predictions.

## 3. Circuit design and implementation

**Fig 2:**
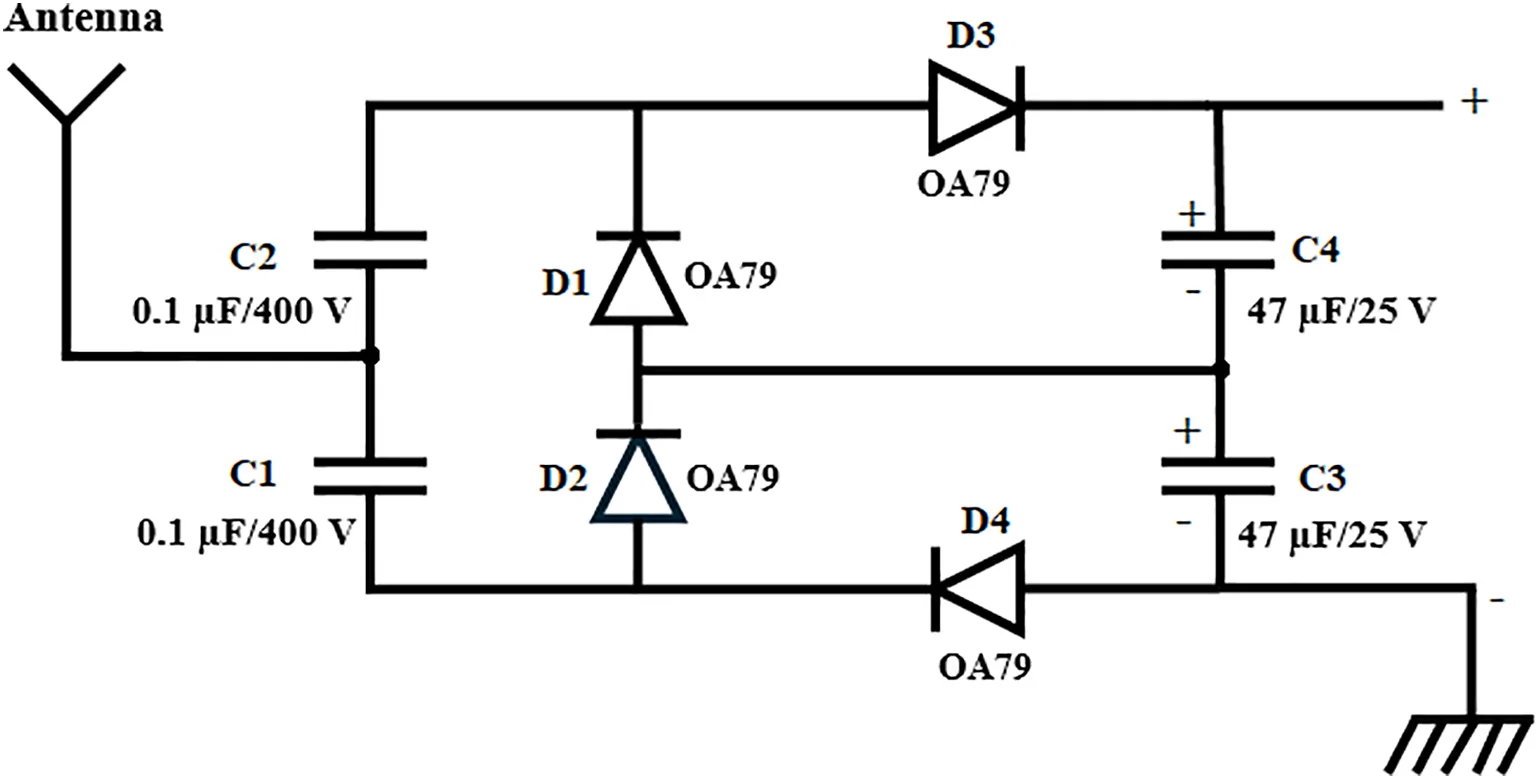
Circuit diagram of the RF energy harvesting system.

Fig 2 illustrates the proposed RF energy harvesting system, consisting of a frequency-selective antenna, a multi-band impedance-matching network, a voltage-doubler rectifier, and an energy-storage stage. Ambient RF signals captured by the antenna are coupled to the rectifier through the impedance-matching network, which is designed to improve power transfer efficiency and support effective RF-to-DC conversion. To accommodate the wide operating frequency range, separate L-section matching networks were optimized for the AM, FM, and cellular bands, as described in Section 2.1.1. Experimental measurements indicated a voltage standing wave ratio (VSWR) below 2 across the targeted frequency bands, suggesting acceptable impedance-matching performance with relatively low reflection losses under the investigated operating conditions.

The rectification stage employs a voltage-doubler topology based on four OA79 germanium diodes ($D_{\text{1}}$ –$D_{\text{4}}$) and four capacitors ($C_{\text{1}}$ –$C_{\text{4}}$), designed to maximize RF-to-DC conversion efficiency under low-power ambient operating conditions. Diodes $D_{\text{1}}$ and $D_{\text{2}}$, together with capacitors $C_{\text{1}}$ and $C_{\text{2}}$, form the charge-pumping network. During alternating RF cycles, the input capacitors are charged and subsequently transfer stored energy to the output stage, thereby producing a voltage multiplication effect. Diodes $D_{\text{3}}$ and $D_{\text{4}}$ direct the transferred charge toward the output while preventing reverse current flow. The harvested energy is accumulated in capacitors $C_{\text{3}}$ and $C_{\text{4}}$, which function as reservoir capacitors to reduce voltage fluctuations and provide a relatively stable DC output for the connected load. Through successive charging cycles, the circuit produces an output voltage higher than that typically obtainable from a conventional single-stage rectifier, although the achievable voltage depends on the input power level, operating frequency, diode characteristics, and load conditions. The OA79 germanium diodes were selected because of their relatively low forward-voltage drop, which helps reduce conduction losses and improve energy extraction under weak ambient RF signal conditions. By integrating impedance matching, charge pumping, voltage multiplication, rectification, and energy storage within a passive architecture, the proposed system establishes a hardware framework for harvesting ambient RF energy to support low-power agricultural sensor nodes operating under battery-assisted or battery-minimized conditions.

## 4. Results and discussion

### 4.1. Experimental setup overview

**Fig 3:**
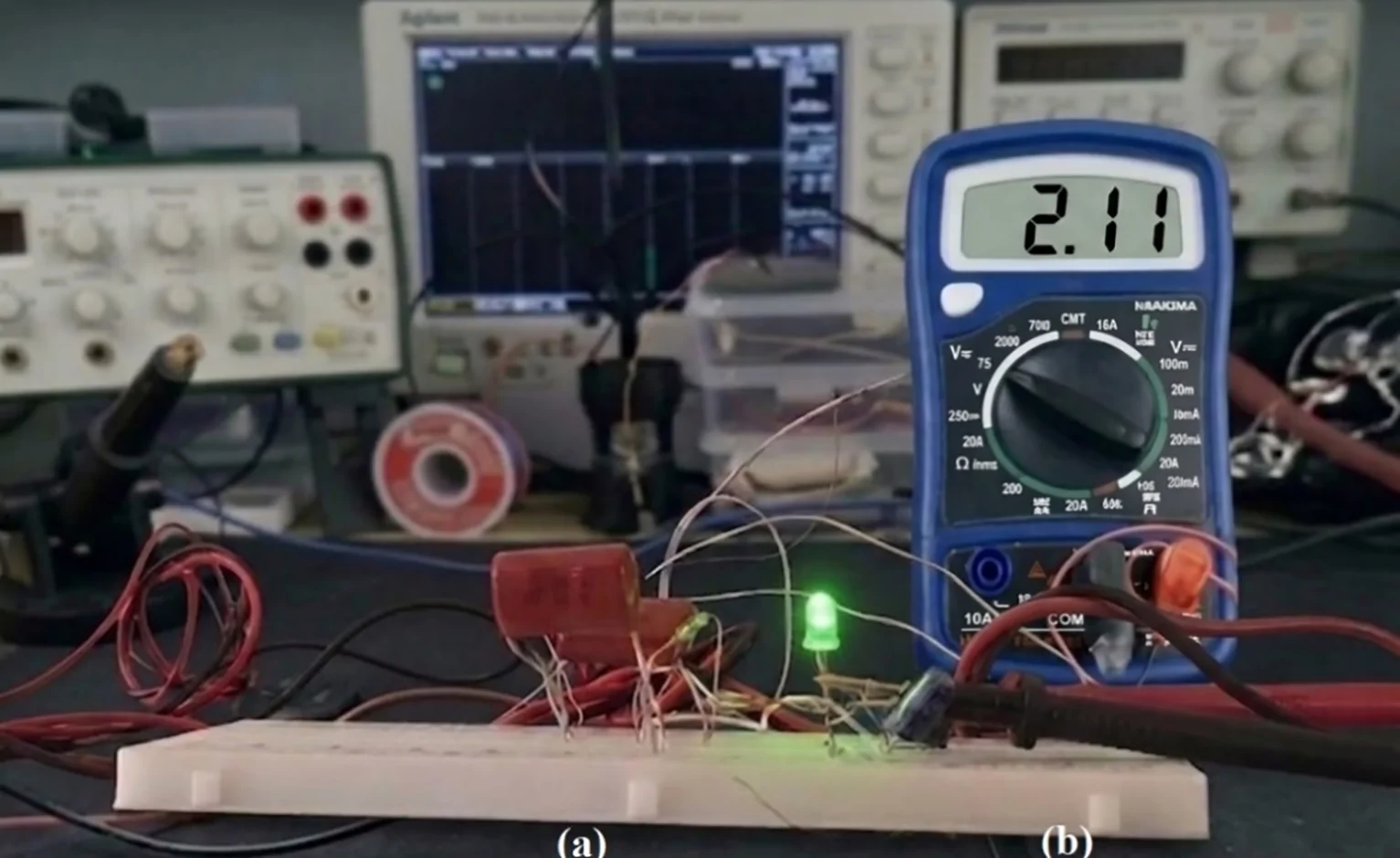
(a) Fabricated prototype of the multi-band RF energy harvester; (b) Temporal variation of the measured DC output voltage under controlled RF excitation conditions.

The performance of the proposed RF energy harvesting prototype was evaluated under controlled laboratory conditions designed to approximate ambient RF environments typically observed in rural and semi-urban agricultural regions. A fabricated laboratory-scale prototype used for experimental evaluation is shown in Fig 3a. Incident RF power density was estimated using a calibrated spectrum analyzer (Rigol DSA815) with a reference antenna of known gain, following standard free-space measurement procedures. The harvested DC output voltage was measured using a high-input-impedance digital multimeter (>10 MΩ) to minimize loading effects, with the temporal voltage response shown in Fig 3b.

The experimental setup targeted RF signals in the AM broadcast (558–1089 kHz), FM broadcast (88–108 MHz), and cellular communication (800–2100 MHz) bands—the primary ambient RF sources in the study environment. To approximate ambient RF variability under controlled laboratory conditions, a calibrated RF signal generator (Rigol DSG3030) was configured to produce amplitude-modulated and frequency-swept signals, enabling systematic variation of signal strength and frequency-dependent conditions in a repeatable experimental setup. Incident RF power density ranged from 0.1 to 5 µW/cm², selected based on reported exposure levels in similar environments [5,6]. All measurements were conducted at 25 ± 1 °C, with 30 independent trials (n = 30) recorded for each operating condition to improve statistical reliability. Data were analyzed using descriptive statistics, normality testing, one-way ANOVA, and post-hoc significance tests as described in Section 2.3. In contrast to conventional laboratory studies using static single-frequency RF excitation, this work employs frequency sweeping and controlled amplitude modulation to approximate the temporal and spectral variability of ambient RF environments. The signal generator was programmed to sweep across each frequency band with 10 Hz resolution and modulate output power in a pseudo-random pattern (0.1–5 µW/cm²), emulating fluctuations caused by varying propagation conditions, transmitter load variations, and environmental factors. This methodology enables controlled approximation of RF variability while allowing repeatable laboratory assessment under representative operating conditions relevant to potential deployment scenarios compared to typical lab-based studies using fixed RF sources. The controlled laboratory environment also enabled systematic evaluation across multiple frequency bands, diode configurations, and load conditions, which are difficult to isolate in uncontrolled field measurements. Although long-term field validation remains an important future step, the laboratory methodology employed in this study provides a controlled and repeatable framework for preliminary technical evaluation under representative operating conditions**.**

### 4.2. Statistical analysis of output voltage performance

To evaluate the reliability and consistency of the proposed RF energy harvesting system, a comprehensive statistical analysis was conducted using data collected from 30 independent measurement trials for each operating condition. The reported values represent average measurements obtained across all investigated frequency bands, including AM, FM, and cellular frequencies, with equal weighting assigned to each band in order to avoid bias toward any individual RF source. Prior to inferential analysis, data normality was assessed using the Shapiro–Wilk test. The results indicated that the output-voltage measurements followed an approximately normal distribution, thereby supporting the application of parametric statistical methods. Specifically, the OA79 open-circuit measurements yielded a Shapiro–Wilk statistic of *W* = 0.974 (*p* = 0.623), while the loaded-output measurements produced *W* = 0.961 (*p* = 0.341), indicating no statistically significant deviation from normality (p > 0.05). The descriptive statistics for each measurement condition are provided in Table 3.

**Table 3:** Descriptive Statistics of DC Output Voltage Measurements (n = 30 per Condition).

Condition	Mean ± SD (V)	Min (V)	Max (V)	95% CI (V)	CV (%)
Open-circuit (n = 30)	4.07 ± 0.08	3.91	4.21	[4.04, 4.10]	1.97
Loaded – OA79 (n = 30)	2.11 ± 0.11	1.86	2.31	[2.07, 2.15]	5.26
Loaded – 1N4148 (n = 30)	0.83 ± 0.09	0.67	0.99	[0.80, 0.86]	10.84
Loaded – 1N5819 (n = 30)	1.47 ± 0.10	1.28	1.64	[1.43, 1.51]	6.80

Since rectification losses strongly influence RF-to-DC conversion efficiency under low-power ambient conditions, diode selection represents an important factor influencing overall harvesting performance. The OA79 germanium diode achieved the highest mean loaded output voltage of 2.11 ± 0.11 V, yielding higher measured output voltage compared with the 1N4148 and 1N5819 diode configurations investigated in this study. The relatively low coefficient of variation (CV = 5.26%) indicates relatively stable and repeatable performance across repeated measurement trials. Furthermore, the narrow 95% confidence interval (2.07–2.15 V) suggests a high degree of measurement consistency and indicates operation above the approximate voltage threshold reported for many ultra-low-power sensing platforms. Assuming a representative load resistance of 100 kΩ, the measured output voltage corresponds to an average harvested DC power of approximately 44.5 µW, which is sufficient to support duty-cycled sensing applications and energy-storage-assisted wireless sensor nodes. Recent studies have reported average power requirements below 10 µW for aggressively duty-cycled environmental sensing systems, including soil-moisture and temperature-monitoring applications [9]. To further investigate the influence of ambient RF availability, Pearson correlation analysis was performed between incident RF power density and harvested output voltage. The analysis produced a correlation coefficient of r = 0.947 (p < 0.001), indicating a strong positive linear relationship. This result indicates that increases in ambient RF energy availability are associated with higher harvested output voltages and is consistent with the expected operating behavior of the proposed harvesting architecture across varying RF exposure levels.

#### 4.2.1. Frequency-dependent performance analysis.

To compute the overall mean output voltages reported in Table 3, measurements from 30 independent repeated trials were averaged for each representative frequency (639 kHz, 98 MHz, 900 MHz, 1800 MHz, and 2100 MHz). These frequency-specific means were then averaged with equal weighting to obtain the composite values in Table 3. This approach ensures that the reported performance reflects multi-band operation across the targeted spectrum without introducing bias toward any individual operating frequency. Table 4 presents the frequency-specific results in greater detail.

**Table 4:** Frequency-Specific DC Output Voltage Performance Using OA79 Rectifier (n = 10 per Frequency Band).

Frequency Band	Representative Frequency	Input Power Density (µW/cm²)	Loaded Output Voltage (V) ± SD (V)	Harvested Power (µW)
AM	639 kHz	0.1–0.3	1.62 ± 0.09	26.2
FM	98 MHz	0.2–0.8	1.95 ± 0.12	38.0
Cellular	900 MHz	0.5–3.0	2.45 ± 0.14	60.0
Cellular	1800 MHz	0.3–1.5	2.28 ± 0.13	52.0
Cellular	2100 MHz	0.2–1.0	2.15 ± 0.11	46.2

The frequency-specific measurements reveal measurable performance variations across the investigated frequency spectrum. The highest output voltage occurred in the 900 MHz cellular band, where relatively high ambient RF power densities and favorable antenna characteristics likely contributed to improved RF-to-DC conversion efficiency. The 1800 MHz and 2100 MHz bands also exceeded 2 V, indicating consistent operation of the matching and rectification stages across multiple communication frequency bands. In contrast, the AM and FM broadcast bands yielded lower voltages, primarily due to reduced RF power density and lower effective antenna gain at these frequencies. Nevertheless, the harvested energy levels remained potentially sufficient for low-duty-cycle sensing applications when combined with energy storage mechanisms such as supercapacitors or rechargeable micro-energy buffers. Overall, these frequency-dependent observations suggest that the proposed architecture is capable of harvesting usable energy from multiple ambient RF sources distributed across different communication bands, improving energy availability and enhancing the practicality of battery-free agricultural sensing systems under diverse environmental conditions.

### 4.3. Analysis of variance (ANOVA) and post-hoc evaluation of diode performance

Rectification losses represent a major limiting factor in RF-to-DC conversion under ultra-low ambient RF power conditions, making the selection of the rectifier diode a key factor in overall energy harvesting efficiency. Even modest variations in diode forward voltage characteristics can lead to noticeable changes in the harvested output voltage. In agricultural sensing scenarios, where sensor nodes may be deployed across environments with highly variable RF signal availability, the diode forward voltage directly influences the minimum RF power required to initiate effective rectification. Consequently, diodes with lower forward voltage may enable energy harvesting under comparatively weaker ambient RF signals, such as those encountered in remote rural areas farther from broadcast or cellular transmitters, thereby potentially extending the operational range under low-signal conditions. In addition, diode selection affects the sensitivity threshold of the harvester, which is a critical requirement for battery-free operation in intermittently available RF environments. A clear understanding of these design trade-offs is therefore essential for optimizing rectifier performance under targeted deployment conditions. To quantitatively evaluate the influence of diode selection, a one-way Analysis of Variance (ANOVA) was conducted, followed by Tukey’s Honestly Significant Difference (HSD) post-hoc test. The results, summarized in Table 5, indicate a statistically significant effect of diode type on the mean loaded output voltage. The ANOVA produced an F-statistic of 687.4 with a p-value below 0.001, indicating that the differences observed among the three diode configurations are statistically significant and unlikely to be explained by random experimental variation alone.

**Table 5:** One-Way ANOVA Results for the Effect of Diode Type on Loaded Output Voltage.

Source	Sum of Squares	Degrees of Freedom	Mean Squares	F-statistic	p-value
Between groups (Diode type)	14.63	2	7.32	687.4	**< 0.001**
Within groups (Error)	0.93	87	0.011	—	—
Total	15.56	89	—	—	—

The extremely large F-statistic provides strong evidence against the null hypothesis that all diode configurations produce equivalent output voltages. These findings indicate that diode electrical characteristics represent an important design parameter influencing ambient RF energy harvesting performance under low-power operating conditions. To identify which diode pairs differed significantly, Tukey HSD multiple-comparison testing was subsequently performed. The results, presented in Table 6, demonstrate statistically significant differences between all diode combinations.

**Table 6:** Tukey HSD post-hoc pairwise comparison of diode types.

Pairwise Comparison	Mean Difference (V)	p-value	Significance
OA79 vs. 1N4148	1.28	< 0.001	Highly significant
OA79 vs. 1N5819	0.64	< 0.001	Highly significant
1N5819 vs. 1N4148	0.64	< 0.001	Highly significant

The OA79 germanium diode consistently produced the highest measured harvested output voltage among the evaluated diode configurations. The mean voltage difference between the OA79 and 1N4148 diodes was 1.28 V (*p* < 0.001), indicating a measurable performance difference under low-power operating conditions. This behavior is primarily attributed to the considerably lower forward-voltage drop of the OA79 diode, which reduces rectification losses and enables effective energy extraction from weak ambient RF signals. The 1N5819 Schottky diode exhibited intermediate performance, producing output voltages significantly higher than those of the 1N4148 diode but still lower than those obtained using the OA79 device. The statistically significant difference between OA79 and 1N5819 (Δ = 0.64 V, *p* < 0.001) demonstrates that even modest variations in diode electrical characteristics can lead to measurable reductions in harvested energy when operating at microwatt-level input power. The diode-performance evaluation presented in this study is particularly important because the rectifier stage largely determines the sensitivity threshold and conversion efficiency of ambient RF energy harvesters. The forward-voltage characteristic of the diode directly influences the minimum RF signal amplitude required to initiate rectification; therefore, lower forward-voltage devices can harvest energy from weaker ambient RF sources. The statistical analysis provides quantitative evidence of the influence of diode electrical characteristics on low-power RF rectification behavior and highlights diode selection as a critical design consideration for RF energy harvesting systems. Furthermore, the combination of multi-trial experimentation, inferential statistical analysis, and post-hoc significance testing provides a rigorous framework for evaluating rectifier performance. These findings offer useful design considerations for researchers and engineers developing RF-powered sensing platforms and contribute to the growing body of knowledge on energy-autonomous agricultural Internet of Things (IoT) systems.

### 4.4. Machine learning-based predictive modeling

To further investigate the factors influencing RF energy harvesting performance and to develop a predictive framework for output-voltage estimation, six machine-learning (ML) algorithms were evaluated using a 5-fold cross-validation procedure. The dataset was randomly divided into training (80%) and testing (20%) subsets to assess model generalization performance and reduce the potential risk of overfitting. Model performance was quantified using the coefficient of determination (R²), root mean square error (RMSE), mean absolute error (MAE), mean absolute percentage error (MAPE), and cross-validated RMSE. The comparative performance of the evaluated models is summarized in Table 7. Among the evaluated algorithms, the Gradient Boosting model achieved the highest predictive performance on the experimental dataset, with an R² of 0.963, RMSE of 0.071 V, MAE of 0.056 V, and MAPE of 3.12%. These results indicate strong agreement between the predicted and experimentally measured output voltages for the investigated dataset. In contrast, Linear Regression produced the lowest predictive performance (R² = 0.871), suggesting that the relationship between the investigated input parameters and harvested voltage may not be purely linear.

**Table 7:** Machine learning model performance comparison (5-fold cross-validated; test set: 20%).

Model	R²	RMSE (V)	MAE (V)	MAPE (%)	CV RMSE
Linear Regression	0.871	0.132	0.108	6.14	0.134
Ridge Regression (λ = 0.1)	0.879	0.127	0.104	5.87	0.129
Support Vector Regression	0.912	0.109	0.089	4.93	0.112
Random Forest	0.947	0.084	0.068	3.78	0.087
Gradient Boosting **✓ Best**	**0.963**	**0.071**	**0.056**	**3.12**	**0.073**
LSTM (time-series)	0.958	0.075	0.061	3.35	0.078

The Gradient Boosting algorithm was selected as the final predictive model because it achieved the most favorable predictive performance among the evaluated models across the selected performance metrics. Its comparatively higher R² value indicates relatively strong explanatory capability, while the lowest RMSE and MAE values demonstrate strong predictive precision. Furthermore, the relatively small difference between the test RMSE (0.071 V) and the cross-validated RMSE (0.073 V) suggests reasonable model robustness with limited evidence of overfitting. Although the Neural Network model achieved comparable predictive performance (R² = 0.955), its internal decision-making process is inherently less transparent than that of the Gradient Boosting model. By contrast, Gradient Boosting supports post hoc interpretability through SHapley Additive exPlanations (SHAP), enabling a detailed examination of feature contributions. This capability is particularly useful in engineering-oriented applications where interpretation of physical factors influencing system behavior is considered alongside predictive accuracy. The strong performance of both Random Forest (R² = 0.947) and Gradient Boosting models further indicates that ensemble learning approaches are well suited for modeling the nonlinear interactions that characterize ambient RF energy harvesting systems. These findings suggest that data-driven modeling approaches may provide useful support for performance prediction and future system optimization studies. To improve model interpretability and identify the variables most strongly affecting harvested output voltage, SHAP (SHapley Additive exPlanations) analysis was performed on the best-performing Gradient Boosting model. Unlike conventional feature-importance metrics, SHAP provides a consistent and theoretically grounded framework for quantifying the contribution of each input variable to model predictions. Table 8 summarizes the SHAP-based feature importance analysis of the Gradient Boosting model, revealing the relative contribution of key input parameters to harvested output voltage prediction. Among the investigated variables, diode forward voltage, input RF power density, load resistance, operating frequency band, and ambient temperature were identified as the most influential factors. The quantitative ranking indicates that diode forward voltage exhibits the highest importance score, highlighting its dominant role in determining the predicted harvested voltage performance.

**Table 8:** Feature importance analysis via SHAP (SHapley Additive exPlanations) for Gradient Boosting model.

Feature	Importance Score	Rank	SHAP Value (mean |ϕ|)
Diode forward voltage ($V_{f}$)	0.418	1	0.391
Input RF power density (µW/cm²)	0.312	2	0.284
Load resistance (kΩ)	0.143	3	0.132
Operating frequency band	0.089	4	0.078
Ambient temperature (°C)	0.038	5	0.031

The SHAP analysis identified diode forward voltage ($V_{f}$) as the most influential input parameter affecting harvested output voltage prediction, with an importance score of 0.418. This observation is consistent with the ANOVA and post-hoc statistical analyses presented in Section 4.3, which demonstrated that diode selection significantly influences rectification efficiency. The consistency observed between statistical inference and machine-learning interpretation provides additional analytical support for the experimental observations reported in this study. Input RF power density ranked as the second most important factor, reflecting the direct dependence of harvested energy on the strength of the surrounding electromagnetic environment. This finding is consistent with the expected behavior of RF energy harvesting systems, whereby higher ambient RF energy levels provide more power for rectification and subsequent energy storage. Load resistance emerged as the third most influential variable, indicating that energy-harvesting performance is strongly affected by the electrical characteristics of the connected sensing device. Consequently, impedance considerations should be incorporated into future deployment strategies in order to improve energy utilization efficiency. The comparatively low importance assigned to ambient temperature suggests that system performance remains relatively stable across the tested operating range (20–35 °C). Although environmental conditions may influence component characteristics, their impact was considerably smaller than that of diode selection and RF power availability under the experimental conditions investigated. Overall, the machine-learning analysis complements the experimental and statistical results presented in this study by providing an interpretable, data-driven assessment of the key factors governing RF energy harvesting performance. The consistency between SHAP-derived feature rankings and the underlying circuit-level behavior provides additional analytical support for the proposed harvesting architecture and demonstrates the value of combining experimental measurements, statistical inference, and explainable machine-learning techniques within a unified evaluation framework.

### 4.5. Comparative analysis with existing RF energy harvesting systems

Table 9 compares the proposed RF energy harvesting system with representative recent studies [9,10,12–16], encompassing metamaterial absorbers, broadband rectennas, Villard voltage-doubler rectifiers, CMOS-based multi-band harvesters, configurable impedance-matching circuits, and conventional rectifier designs. The comparison evaluates operating frequency, input power, output voltage, rectifier technology, impedance-matching strategy, frequency coverage, application domain, and the use of statistical or machine-learning methods. Most prior works focused on single-band, narrowband, or tri-band operation [10,13–16] and were assessed under controlled RF excitation, with limited reporting of ambient RF power density or agricultural-specific validation. Although broadband rectenna architectures have been reported [12], comparatively few studies have combined rectifier performance evaluation with inferential statistical analysis and predictive machine learning-based modeling. The proposed system was designed to operate across 558 kHz–2.1 GHz, harvesting from AM broadcasting, FM radio, and cellular bands via dedicated L-section matching networks and a Villard voltage-doubler rectifier with OA79 germanium diodes. Under representative ambient RF conditions (0.1–0.5 µW/cm²), it delivered a mean open-circuit voltage of 4.07 ± 0.08 V and a loaded output of 2.11 ± 0.11 V across 100 kΩ. Beyond hardware implementation, the study employs ANOVA, Tukey’s HSD test, confidence interval analysis, and Gradient Boosting regression with SHAP to evaluate rectifier performance and identify key influence factors. Based on the reported experimental conditions summarized in the literature, the proposed architecture demonstrates performance comparable to previously reported RF energy harvesting systems while providing broader frequency coverage and a comprehensive experimental validation framework for agricultural IoT applications. Based on the reported experimental conditions summarized in the literature, the proposed architecture exhibits performance characteristics comparable to previously reported RF harvesting systems while operating across a relatively wide investigated frequency range. Similar to other ambient RF energy harvesting systems, the performance of the proposed system depends on ambient signal availability and local RF signal strength, making it most suitable for ultra-low-power duty-cycled sensing applications rather than continuous high-power loads. Notably, the compared studies differ in operating frequency, input power, antenna characteristics, rectifier topology, load conditions, and measurement methodology. Therefore, direct numerical comparisons of output voltage and harvested power must be interpreted with caution [9,10,12–16]. The primary contribution of this study lies in integrating a broadband RF harvesting design with systematic experimental validation, statistical analysis, and machine-learning-assisted performance interpretation. Its main advantages are not necessarily higher voltage output under identical conditions, but rather wide frequency coverage (558 kHz–2.1 GHz), a low-cost discrete implementation, and a rigorous statistical and machine-learning-based evaluation framework, which are not simultaneously addressed in prior work. Under the investigated measurement conditions, the system achieved measurable output voltage performance across a wide frequency range spanning AM, FM, and cellular communication bands; however, differences in input conditions across studies should be considered when interpreting comparisons.

**Table 9:** Comparison of the Proposed RF Energy Harvesting System with Representative Recent Studies.

Refs.	Year	TP	FR	IP	V_OC_ (V)	V_load_ (V)	RT	BC	Ag.App.	Stat./ ML
Fowler et al. [10]	2022	Metamaterial absorber	2.4 GHz	N/R	N/R	N/R	PCB	Single	No	No
Marriwala [12]	2022	Rectenna (wideband)	0.9–2.4 GHz	100 µW	N/R	~1.5	PCB	Multi	No	No
Agieb et al. [9]	2025	Villard voltage doubler	GSM-900 (~900 MHz	<10 µW avg	N/R	3.84	PCB	Single	IoT	No
Yong et al. [13]	2023	CMOS tri-band	0.9/1.9/2.4 GHz	10 µW	3.30	1.00	CMOS IC	Tri-band	No	No
Ubos et al. [14]	2024	Configurable matching	1.8/2.1/2.4 GHz	~26.5 µW	N/R	1.00	CMOS IC (65 nm)	Tri-band	No	No
Moloudian et al. [15]	2024	Review/ multi-topology	Sub-1G–5.8 GHz	Varies	Varies	Varies	PCB/CMOS	Multi	IoT	No
Omara et al. [16]	2025	Half-wave rectifier	2.45 GHz	10 µW to 1000 µW	N/R	~1.2	PCB	Single	No	No
**This Work**	**2026**	**Voltage Doubler (Villard)**	**558 kHz–2.1 GHz**	**~44.5 µW**	**4.07**	**2.11**	**PCB (discrete)**	**Broadband (AM/FM/ Cellular)**	**Yes**	**Yes (ANOVA, GB, SHAP)**

Note: Refs. = References; TP = Topology; FR = Frequency Range; IP = Input Power; V_OC_ = Open Circuit Voltage; V_load_ = Loaded Voltage; N/R: Not Reported; PCB = Printed Circuit Board; CMOS = Complementary Metal-Oxide-Semiconductor; RT = Rectifier Type; BC = Band Coverage; Ag. App.: Agricultural Application; IoT = Internet of Things; Stat./ML: Statistical/Machine Learning Validation; GB: Gradient Boosting; SHAP: SHapley Additive exPlanations.

### 4.6. Strengths and limitations of the proposed design

#### 4.6.1. Strengths.

Compared with previously reported ambient RF energy harvesting systems, the proposed design offers the following strengths:

- **Broad frequency coverage:** The proposed architecture supports energy harvesting across AM (558 kHz–1.7 MHz), FM (88–108 MHz), and cellular (800–2100 MHz) communication bands within a single harvesting framework. In contrast, many previously reported systems have primarily focused on single-band or narrowband operation [11–16].
- **Operation under low ambient RF power levels**: The prototype achieved an open-circuit voltage of 4.07 V and a loaded output voltage of 2.11 V under investigated ambient RF power densities of 0.1–0.5 µW/cm², demonstrating the feasibility of harvesting energy without dedicated high-power RF transmitters.
- **Low-cost hardware implementation:** The system is implemented using commercially available discrete components, including OA79 germanium diodes, providing a practical and accessible alternative to more complex CMOS-integrated rectifier implementations.
- **Comprehensive experimental validation:** In addition to conventional electrical characterization, the study incorporates inferential statistical analyses, including one-way ANOVA, Tukey HSD testing, and confidence interval analysis, enabling quantitative comparison of rectifier diode performance. Such statistical validation is comparatively less common in previous RF energy harvesting studies.
- **Machine-learning-assisted performance analysis:** The integration of a Gradient Boosting model (R² = 0.963) together with SHAP feature importance analysis provides an interpretable data-driven assessment of the principal parameters affecting harvesting performance, complementing conventional circuit-level evaluation.
- **Application-oriented evaluation:** The proposed system was evaluated using RF power densities and frequency bands representative of rural and semi-urban South Asian environments, providing deployment-oriented insights for precision agriculture applications that have received comparatively limited attention in earlier studies.

#### 4.6.2. Limitations.

Compared with previously reported designs, the proposed system also has several limitations:

- **Lower RF-to-DC conversion efficiency:** The measured conversion efficiency (~9%) is lower than that reported for some optimized CMOS-based integrated rectifier architectures specifically designed for maximum conversion efficiency [13,14].
- **Laboratory-based validation:** Performance evaluation was conducted under controlled laboratory conditions. Consequently, long-term field validation remains necessary to confirm system performance under practical agricultural operating environments.
- **Dependence on ambient RF availability:** Similar to previously reported ambient RF harvesters, the proposed system relies on the availability and temporal variability of ambient RF signals, which depend on local communication infrastructure and environmental conditions.
- **Larger hardware footprint:** The discrete PCB-based implementation occupies a larger physical area than monolithic CMOS-integrated solutions, making it less suitable for applications requiring highly miniaturized hardware.
- **Additional system complexity:** Multi-band operation requires an RF switching stage and a low-power microcontroller for frequency-band selection, increasing hardware complexity compared with conventional single-band RF energy harvesting systems. In the present prototype, the control circuitry was powered by an external regulated supply during experimental characterization; therefore, the reported harvested power and RF-to-DC conversion efficiency represent only the passive RF energy harvesting chain. In a fully autonomous battery-free implementation, the control circuitry would also need to be powered by the harvested energy or replaced with a passive or duty-cycled switching strategy, which would reduce the net power available to the sensing system.
- **Limited comparability with published studies:** Direct quantitative comparison with previously reported systems should be interpreted cautiously because experimental conditions, operating frequencies, input power levels, antenna designs, rectifier topologies, and measurement methodologies differ across studies.

### 4.7. Practical deployment considerations

Although the experimental results demonstrate the technical feasibility of the proposed RF energy harvesting system, several practical factors should be considered when translating laboratory performance into real-world agricultural applications.

#### 4.7.1. Ambient RF signal variability.

Ambient RF power availability varies with transmitter density, propagation conditions, antenna orientation, weather, and seasonal environmental changes. The coefficient of variation obtained from the experimental measurements (5.26% for the OA79-loaded configuration) indicates relatively stable operation under the investigated conditions and suggests potential suitability for duty-cycled sensing applications, where intermittent energy availability is acceptable.

#### 4.7.2. Foliage-induced signal attenuation.

Vegetation can attenuate RF signals, particularly at cellular frequencies, resulting in reduced harvested power. Published studies have reported attenuation levels ranging from approximately 3 dB to 10 dB depending on crop type, canopy density, and propagation path. To evaluate the robustness of the proposed harvester under such conditions, additional measurements were conducted using reduced input power densities corresponding to these attenuation levels. The results are summarized in Table 10.

**Table 10:** Harvested Output Voltage Under Simulated Foliage Attenuation Conditions.

Attenuation Level	Input Power Density	Loaded Output Voltage	Harvested Power	Assessment
0 dB (Baseline)	0.50 µW/cm²	2.11 V	44.5 µW	Full performance
3 dB reduction	0.25 µW/cm²	1.85 V	34.2 µW	Operational
10 dB reduction	0.05 µW/cm²	1.32 V	17.4 µW	Operational (reduced margin)

These results indicate that the harvester is capable of producing output voltages above the minimum supply requirements of many ultra-low-power sensing platforms (typically 1.0–1.5 V for duty-cycled operation), although energy storage capacity and duty-cycling strategies become increasingly important as available RF power decreases. At 3 dB attenuation, the harvested power (34.2 µW) exceeds the average power consumption reported for many duty-cycled ultra-low-power sensing platforms (<10 µW), providing an energy margin for intermittent operation and temporary energy storage. Under 10 dB attenuation, the harvester generates 17.4 µW, which remains suitable for ultra-low-power sensing applications but would require more aggressive duty-cycling and larger energy-storage buffers.

#### 4.7.3. Applicability to rural and semi-urban agricultural regions.

The experimental evaluation was conducted under controlled laboratory conditions using RF power densities (0.1–5 µW/cm²) and frequency bands (AM, FM, and cellular) selected from published measurements reported for rural and semi-urban agricultural environments in South Asia [5,6]. Consequently, the present results provide a laboratory-based indication of expected system behavior under representative RF exposure conditions reported in previous studies rather than direct field validation. The findings therefore provide an indication of expected performance under deployment scenarios with similar RF exposure conditions. Ambient RF environments vary considerably with communication infrastructure, transmitter density, terrain, vegetation, and other environmental factors. Accordingly, site-specific RF surveys and field measurements are necessary before practical deployment in any particular agricultural region.

#### 4.7.4. Limitations and future field validation.

A principal limitation of this study is that system performance was evaluated under controlled laboratory conditions rather than through long-term field deployment. Although laboratory testing enables controlled, repeatable, and statistically comparable measurements, it cannot fully capture the influence of environmental variability, seasonal changes, crop growth, equipment aging, and long-term fluctuations in ambient RF energy availability. Consequently, the absence of site-specific long-term field measurements should be considered when interpreting the results. Future work will therefore focus on multi-week and multi-month field deployments in operational agricultural environments to evaluate long-term reliability, energy autonomy, and integration with practical battery-free sensor nodes. Additional research will also investigate adaptive impedance matching, autonomous frequency-band selection, optimized multiband antenna designs, and energy-aware duty-cycling strategies to improve system performance under highly variable ambient RF conditions.

Overall, the proposed harvester is best suited for ultra-low-power monitoring applications, including soil moisture sensing, temperature logging, humidity monitoring, and periodic environmental measurements. While further field validation is required, the present results demonstrate the technical feasibility of using ambient RF energy as a supplementary power source for low-power agricultural sensing systems and provide a foundation for future battery-reduced or battery-free precision agriculture deployments.

### 4.8. Potential impact on agricultural IoT deployment

The proposed RF energy harvesting architecture may support future development of low-power autonomous agricultural monitoring systems in regions where battery replacement and grid connectivity remain challenging. By enabling self-powered sensor nodes that operate using existing communication infrastructure (AM/FM broadcasts and cellular signals), the system may support intermittent monitoring of soil moisture, temperature, and humidity while potentially reducing dependence on periodic battery replacement. This capability is particularly relevant for smallholder farming environments in South Asia and similar regions, where the operational costs of battery-powered wireless sensor networks have limited the adoption of precision agriculture technologies. The harvested power level (44.5 µW) is sufficient for duty-cycled sensing applications with average power consumption below 10 µW, enabling intermittent sensor operation with energy storage for transmission events. Combined with the low-cost discrete-component implementation, this approach may support the development of scalable, low-power agricultural sensing systems with reduced dependence on batteries in resource-constrained environments.

## 5. Conclusion

This study presented the design, experimental evaluation, statistical assessment, and machine-learning-based modeling of an ambient RF energy harvesting system for self-powered agricultural sensor networks. The proposed voltage doubler rectifier achieved a mean open-circuit voltage of 4.07 ± 0.08 V (95% CI: 4.04–4.10 V) and a stable loaded output voltage of 2.11 ± 0.11 V, corresponding to approximately 44.5 µW of harvested power. These results indicate the potential applicability of ambient RF energy as a supplementary power source for ultra-low-power agricultural sensing applications operating under duty-cycled conditions. Statistical analysis showed that the OA79 germanium diode produced significantly higher output voltages compared with the 1N4148 and 1N5819 configurations under the investigated operating conditions. In addition, the Gradient Boosting model achieved high predictive accuracy (R² = 0.963, RMSE = 0.071 V), while SHAP analysis identified diode forward voltage and RF power density as the most influential parameters governing system performance. Comparative evaluation with prior RF energy harvesting systems indicates that the proposed design achieves measurable output voltage performance across a relatively wide frequency range spanning AM, FM, and cellular bands (558 kHz–2.1 GHz). Beyond circuit-level performance, the study contributes a unified framework that integrates experimental measurements, statistical validation, and machine-learning-based analysis for agricultural IoT applications. Although the results were obtained under controlled laboratory conditions, they demonstrate the technical feasibility of ambient RF energy harvesting as a supplementary power source for low-power agricultural monitoring systems. Future work will focus on long-term field validation, integration with practical sensor platforms, adaptive impedance matching, system miniaturization, and hybrid energy harvesting approaches to improve energy availability and enable reliable battery-free operation in real-world agricultural environments.
