# Supplementary material for: Ambient RF energy harvesting using voltage doubler rectifier for battery-free agricultural sensor networks: Design, statistical analysis, and machine learning validation
Source: PLoS One. 2026 Sep 10;21(9):e0350236. doi: 10.1371/journal.pone.0350236 (PMC13561346; doi:10.1371/journal.pone.0350236)
Supplement: Supplementary file 1 [file pone.0350236.s001.docx]

**Response to Reviewers**

**Manuscript ID**: PONE-D-26-23129

**Title:** Ambient RF Energy Harvesting Using Voltage Doubler Rectifier for Battery-Free Agricultural Sensor Networks: Design, Statistical Analysis, and Machine Learning Validation

**Authors:** Md. Atik Hasan Nishat, Prithwiraj Biswas Pallab, Nowrin Jannat, Saleha Nasrin Mishu, Md Fahad Ullah Utsho, Md. Bipul Islam, Riaz Uddin Mondal, Md. Firoz Ahmed, M. Hasnat Kabir

Dear Editor-in-Chief,

We sincerely thank the Academic Editor and both reviewers for their thorough and constructive evaluation of our manuscript. Their insightful comments and recommendations have substantially improved the quality, clarity, and rigor of our work. Below, we provide a detailed, point-by-point response to each comment raised during the review process. All changes made in the revised manuscript have been highlighted **in red.**

**Response to Journal Requirements**

**1. PLOS ONE style requirements:**

**Response:** We have carefully reviewed and formatted the manuscript according to the PLOS ONE style templates, ensuring compliance with all file naming and formatting guidelines.

**2. ORCID iD validation:**

**Response:** The corresponding author (Dr. Md. Firoz Ahmed) has an ORCID iD, and it has been successfully validated in the Editorial Manager system.

**3. Code sharing guidelines:**

**Response:** We acknowledge PLOS ONE’s guidelines on code sharing. In this study, the machine learning and statistical analyses were performed using standard, widely available open-source libraries (e.g., scikit-learn, XGBoost, SHAP, and standard scientific computing tools in Python/R). No custom, author-generated proprietary code underpins the core findings; therefore, specific code deposition is not applicable. However, the experimental data and analytical workflows are available upon request as detailed in the Data Availability Statement.

**4. Data Availability Statement update:**

**Response:** We have updated the Data Availability Statement to include the specific contact details for data requests. It now reads: *“The experimental data supporting the findings of this study are available upon reasonable request. For institutional oversight and long-term data stewardship, data requests should be directed to the Department of Information and Communication Engineering, University of Rajshahi, Rajshahi-6205, Bangladesh (Email: ice@ru.ac.bd), which serves as the primary institutional non-author point of contact for managing data requests. Requests may also be directed to the corresponding author, Dr. Md. Firoz Ahmed (Email: firozice01@ru.ac.bd). The experimental measurement data contain no personally identifiable information or other ethical restrictions and are available for verification and reproducibility purposes upon reasonable request.”*

**5. Reference to Figure 3:**

**Response:** We have ensured that Figure 3 is explicitly referenced in the text (Section 4.1, paragraph 1) as required for production linking.

**6. Review of recommended citations:**

**Response:** We thank the editor for pointing this out. We have carefully reviewed the recommended publications and found them highly relevant to our work on multi-band RF energy harvesting. We have incorporated citations for the suggested multi-band/tri-band works by Ubos et al. and Yong et al. into the manuscript (now References [13] and [14]) and included them in our comparative analysis.

**Response to Reviewer #1**

We thank the reviewer for their positive assessment of our work and for recognizing the significance and comprehensive nature of our study. We have addressed the suggested corrections as follows:

**Comment a:** *The comparison table needs to include some recent state-of-the-art literature in chronological order (mention the year in the corresponding references).*

**Response:** We agree with this suggestion. Table 8 has been updated to include recent state-of-the-art literature (spanning from 2022 to 2026). A dedicated “Year” column has been added, and the references are now arranged in chronological order to provide a clear timeline of technological advancements in this field.

**Comment b:** *Highlight the pros and cons of the proposed design in bulleted form over the ones reported earlier.*

**Response:** We have added a new subsection (Section 4.6) that explicitly outlines the strengths and limitations of our proposed design in a clear, bulleted format. Section 4.6.1 details the pros (e.g., broad frequency coverage, low-cost hardware, comprehensive statistical validation), while Section 4.6.2 addresses the cons (e.g., lower conversion efficiency compared to CMOS, larger hardware footprint).

**Comment c:** *Do include additional insights regarding its novelty and application usage, which can benefit the upcoming readers in this particular field of interest.*

**Response:** We have added Section 4.8, "Potential Impact on Agricultural IoT Deployment," to highlight the practical novelty and application usage of the proposed system. This section discusses how the harvester can support self-powered sensor nodes for smallholder farming environments in South Asia, reducing dependence on battery replacement and enabling scalable precision agriculture.

**Response to Reviewer #2**

We thank the reviewer for their critical and detailed evaluation, which has helped us significantly clarify our methodology, address ambiguities, and strengthen the manuscript's technical rigor.

**Comment 1:** *Please provide clear citations on the typical ambient RF power densities figure presented in the introduction.*

**Response:** We have added appropriate citations [5–8] in the Introduction to support the stated typical ambient RF power density ranges (0.1–10 µW/cm² in rural environments and higher in semi-urban areas).

**Comment 2:** *In the introduction, the authors make the point that existing studies perform measurements/tests in controlled laboratory environments as a critical research gap, however in their own research findings, the results published are also performed under laboratory conditions. Either this research gap should be removed from the introduction, or authors should clarify why they were unable to provide real world measurement results which could fill the aforementioned research gap.*

**Response:** We appreciate this valid observation. We have clarified this point in both the Introduction and Section 4.1. We explained that while long-term outdoor field measurements are difficult to control and isolate due to extreme environmental variability, our laboratory setup was specifically designed to emulate realistic ambient RF variability using multi-frequency excitation and controlled time-varying power levels (frequency sweeping and pseudo-random amplitude modulation). This allows for repeatable, statistically rigorous experimentation that approximates real-world fluctuations, serving as a necessary foundational step before long-term field deployment.

**Comment 3:** *The authors indicate the multiband (0.5GHz to 2.1GHz) operation as a key functionality of the proposed system, with clear explanation on how its achieved on the antenna side (multiplexer), but none for the Impedance Matching Network. It is stated that “. network consists of an L-section (inductor-capacitor) optimized for each sub-band, achieving a VSWR of <2 across all target frequencies.” Considering that reactance variation from even just slight frequency shifts can cause significant mismatch, more elaboration is required on how the implemented L-matching network is able to cope with such a wide operating frequency. Additional clarification is also needed on whether the multi-band operation is tuned for a few key frequencies eg. 558MHz, 1089MHz, etc. OR for a wide-band approach where matching (VSWR<2) is achieved across the whole 0.5GHz to 2.1GHz spectrum.*

**Response:** We have significantly expanded Section 2.1.1 to clarify this. We explicitly state that instead of using a single broadband matching network (which suffers from severe reactance variations over ultra-wide ranges), we implemented three dedicated, switchable L-section impedance-matching networks. Each network is independently optimized for a specific sub-band (AM, FM, and Cellular). An RF switch, controlled by a microcontroller, selects the appropriate branch based on signal strength. Thus, the system achieves VSWR < 2 across the entire spectrum by tuning discrete narrow-to-mid bands sequentially, rather than attempting a single wide-band match.

**Comment 4:** *Again, please provide clear citations on where the figures for typical RF Power densities in ambient conditions are obtained (Section 2.2).*

**Response:** We have added citations [5–8] in Section 2.2 to justify the selected RF power density range (0.1–5 µW/cm²) based on previous measurement studies in comparable South Asian environments.

**Comment 5:** *Related to point 3, in Section 4, only one result is presented (mean output Voltage), despite the system being presented as a multiband. Please clarify how this presented mean is being calculated. A separate table/graph should also be included to present the results (VOC etc.) at/across all measured frequency points.*

**Response:** We have added Section 4.2.1, **“Frequency-Dependent Performance Analysis,”** to explain that the overall mean is calculated by equally weighting the means of representative frequencies across the bands. Furthermore, we have added **Table 3a,** which explicitly presents the loaded output voltage and harvested power at each specific measured frequency point (639 kHz, 98 MHz, 900 MHz, 1800 MHz, and 2100 MHz).

**Comment 6:** *Provide citations for the <10µW average power draw of duty-cycled sensors figure stated in Section 4.2.*

**Response:** We have added Reference [9] in Section 4.2 to support the claim regarding the average power requirements of aggressively duty-cycled environmental sensing systems.

**Comment 7:** *Fix the formatting for Table 4 and 6 (missing outlines).*

**Response:** The formatting for Table 4 and Table 6 has been corrected, and all table outlines/borders are now properly included in the revised manuscript.

**Comment 8:** *Define the “abbreviation” ANOVA on first use.*

**Response:** We have defined "Analysis of Variance (ANOVA)" upon its first use in both the Abstract and the Introduction.

**Comment 9:** *Additional justification/explanation should be given at the start of Section 5 [now Section 4.3] to highlight the significance of the Diode Performance analysis. Its current inclusion in the manuscript seems slightly out of place.*

**Response:** We have rewritten the introductory paragraph of Section 4.3 to clearly justify the importance of diode selection. We now explain that under ultra-low ambient RF conditions, rectification losses are the primary bottleneck, and the diode's forward voltage directly dictates the minimum sensitivity threshold of the harvester. This contextualizes why a rigorous statistical comparison of diodes is critical for agricultural deployments in remote, weak-signal areas.

**Comment 10:** *The parameters in the comparison table is lackluster and does not provide the readers with sufficient information to make informed judgement of the performance variation across compared works. At minimum, input power, rectifier type (on-chip, pcb etc.), should be stated. If possible, select other multi-band works to compare results. A few relevant multi-band (tri-band) works are listed below:*

*• N. J. Ubos, M. R. Zablan, K. D. Acas, O. J. Gerasta, G. F. Palencia, N. Mapula, K. Maglinte, R. Sabarillo, and J. L. Balassa, ‘‘RF-to-DC conversion circuit with configurable impedance match ing circuit for RF energy harvesting in 65nm CMOS technology,’’ in Proc. TENCON IEEE Region Conf. (TENCON), Singapore, Dec. 2024, pp. 1314–1318.*

*• J. K. Yong, W. X. Lian, H. Ramiah, K. K. P. Churchill, G. Chong, N. S. Lai, Y. Chen, P.-I. Mak, and R. P. Martins, ‘‘A fully integrated CMOS tri-band ambient RF energy harvesting system for IoT devices,’’ IEEE Trans. Circuits Syst. I, Reg. Papers, vol. 70, no. 12, pp. 4705–4718, Dec. 2023*.

**Response:** We have completely overhauled Table 8. We added columns for Input Power Density (IPD), Input Power (IP), Rectifier Type (RT - specifying PCB discrete vs. CMOS IC), and Band Coverage (BC). We have also included the highly relevant multi-band/tri-band works suggested by the reviewer (Yong et al. [13] and Ubos et al. [14]) to provide a much more informed and comprehensive comparison.

**Comment 11:** *Again, please show the results at each/fixed frequencies instead of mean when presenting for comparisons.*

**Response:** As addressed in Comment 5, we have added Table 3a to show the exact results at each fixed frequency. In the revised comparison discussion (Section 4.5), we now explicitly caution readers against direct numerical comparisons of our overall mean with other works' single-frequency results, and instead direct readers to Table 3a for our frequency-specific performance metrics.

**Comment 12:** *Without comparing input conditions, the statement, “The proposed system delivers the highest broadband operational frequency coverage (558 kHz–2.1 GHz), the highest open circuit voltage (4.07 V), and the highest loaded output voltage (2.11 V) among the compared designs.”, does not hold water and should be expanded on.*

**Response:** We completely agree. We have revised this statement in Section 4.5. We now explicitly state that because the compared studies differ vastly in input power, antenna characteristics, and measurement methodologies, direct numerical comparisons of output voltage must be interpreted with caution. We have reframed the contribution to emphasize that our main advantage lies in the combination of ultra-wide frequency coverage, low-cost discrete implementation, and a rigorous statistical/ML validation framework, rather than claiming absolute superiority in voltage output under mismatched input conditions.

**Comment 13:** *Measurement results of the lower input power conditions (3db-10db lower) should be presented to back the claim of the 2V loaded being sufficient to overcome the “Foliage Attenuation” stated in Section 4.6 [now 4.7.2].*

**Response:** We have added a new subsection, Section 4.7.2 **“Foliage-Induced Signal Attenuation,”** which includes **Table 9.** This table presents the measured loaded output voltage and harvested power under simulated foliage attenuation conditions (0 dB baseline, 3 dB reduction, and 10 dB reduction), proving that the harvester remains operational and above the minimum supply thresholds for ultra-low-power sensors even under severe attenuation.

**Comment 14:** “*Geographic Relevance” in Section 4.6 is not a relevant claim as the measurements were still conducted in a laboratory environment. Either remove or reword this statement.*

**Response:** We have removed the term “Geographic Relevance.” The section has been reworded and renamed to **“Applicability to Rural and Semi-Urban Agricultural Regions” (Section 4.7.3**). We explicitly clarify that the laboratory evaluation was designed using RF power densities representative of literature-reported conditions in these regions, providing a laboratory-based indication of expected behavior rather than claiming direct geographic field validation.

**Response to Specific Editorial Team Comments**

**Comment 1:** *Please ensure that you refer to Figure 1 in your text as, if accepted, production will need this reference to link the reader to the figure.*

**Author Response:** We appreciate the editorial team’s observation and have addressed this issue by incorporating an explicit reference to Figure 1 in the revised manuscript.

**Revision Made:** A reference to Figure 1 has been added in **Section 2.1 (*System Architecture Overview*)**. The following sentence has been included:

***“****Figure 1 illustrates the block diagram of the proposed RF energy harvesting system, depicting the signal flow from RF energy capture through impedance matching, rectification, and energy storage stages. The architecture demonstrates the conversion pathway of ambient RF signals into usable DC power for supplying low-power agricultural sensor nodes****.”***

**Location in Revised Manuscript:** Section 2.1, Paragraph 2.

**Evidence of Change:** The citation of Figure 1 has been incorporated into the main text before the figure placement, enabling appropriate cross-referencing and production linking.

**Comment 2:** *Please ensure that Table 7 is referenced in the main text, as this reference is required during production to enable linking between the text and the table.*

**Author Response:** We thank the editorial team for highlighting this requirement. A reference to Table 7 has been added in the revised manuscript to ensure proper linkage between the discussion and the corresponding table.

**Revision Made:** A reference to Table 7 has been incorporated in **Section 4.4 (*Machine Learning-Based Predictive Modeling*)** before the SHAP-based feature importance discussion. The following text has been added:

*“Table 7 summarizes the SHAP-based feature importance analysis of the Gradient Boosting model, presenting the relative contribution of the investigated input parameters to harvested output voltage prediction. The analysis identifies diode forward voltage, input RF power density, load resistance, operating frequency band, and ambient temperature as the dominant contributing factors, with diode forward voltage exhibiting the highest importance score.”*

**Location in Revised Manuscript:** Section 4.4, Paragraph 6.

**Evidence of Change:** The reference to Table 7 has been added within the relevant discussion section, and the table caption has been revised to provide a clearer description of the SHAP-based feature ranking methodology.

We believe that these revisions comprehensively address all the concerns raised by the reviewers and the editorial team. We hope the revised manuscript is now suitable for publication in *PLOS ONE.*

Thank you again for your time and consideration.

Sincerely Yours,

Dr. Md. Firoz Ahmed

On behalf of all authors

Department of Information and Communication Engineering

University of Rajshahi, Rajshahi-6205, Bangladesh

Email: firozice01@ru.ac.bd
