# Supplementary material for: Ambient RF energy harvesting using voltage doubler rectifier for battery-free agricultural sensor networks: Design, statistical analysis, and machine learning validation
Source: PLoS One. 2026 Sep 10;21(9):e0350236. doi: 10.1371/journal.pone.0350236 (PMC13561346; doi:10.1371/journal.pone.0350236)
Supplement: Supplementary file 2 [file pone.0350236.s002.docx]

**Response to Reviewers**

**Manuscript ID:** PONE-D-26-23129R1

**Title:** *Ambient RF Energy Harvesting Using Voltage Doubler Rectifier for Battery-Free Agricultural Sensor Networks: Design, Statistical Analysis, and Machine Learning Validation*
**Journal:** *PLOS ONE*

Dear Editor-in-Chief,

We sincerely thank you and the reviewers for your careful evaluation of our manuscript and for your constructive comments and valuable suggestions. We greatly appreciate the time and effort devoted to reviewing our work. The comments have helped us improve the clarity, transparency, and overall quality of the manuscript. We have carefully considered all comments and revised the manuscript accordingly. A detailed point-by-point response to each comment has been prepared and submitted as a separate **Response to Reviewers** document. For ease of evaluation, all revisions in the marked-up manuscript have been highlighted in red.

**Reviewer #1**

**Comment:** *The reviewer considered the manuscript interesting and significant, noted that it is well written, and indicated that all previous concerns had been satisfactorily addressed.*

**Response:** We sincerely thank Reviewer #1 for the positive assessment and encouraging comments. We greatly appreciate the reviewer's recognition that the concerns raised during the previous review rounds have been satisfactorily addressed. We are grateful for the constructive feedback provided throughout the review process, which has substantially improved the quality of our manuscript.

**Reviewer #2**

**Comment 1:** *The Gemini watermark is present in Figures 1 and 2, indicating that the figures were AI generated or altered. Please remove or replace the affected figures and provide an appropriate disclosure regarding AI-assisted technologies.*

**Response:** We thank the reviewer for identifying this important issue. Figures 1 and 2 have been completely redrawn by the authors using Microsoft Word 2016. The previous figures containing the Gemini watermark have been removed entirely and replaced with original figures prepared by the authors. Consequently, no AI-generated or AI-modified figures are included in the revised manuscript. The **Declaration of AI-Assisted Technologies** has also been revised and now states:

*The authors used ChatGPT solely for language editing and grammatical refinement of the manuscript. All aspects of the study, including the research design, experimental implementation, data collection, statistical analysis, interpretation of the results, preparation of figures, and scientific conclusions, were performed independently by the authors. No generative AI image tools were used in the preparation of the figures included in this manuscript.*

We believe these revisions fully address the reviewer's concern regarding transparency and compliance with the journal's policy on AI-assisted technologies.

**Revision made**

- Figures 1 and 2 were completely redrawn by the authors.
- The Gemini watermarks were removed.
- The Declaration of AI-Assisted Technologies was revised to improve transparency regarding the use of AI-assisted technologies.

**Comment 2:** *Please clarify whether the reported output power and RF-to-DC conversion efficiency include the power consumption of the RF switch and microcontroller used for frequency-band selection.*

**Response:** We appreciate this valuable suggestion. Section 2.1.1 has been revised to explicitly clarify that the RF switch and microcontroller were powered by an external regulated laboratory power supply during experimental characterization. Consequently, the reported open-circuit voltage, loaded output voltage, harvested power, and RF-to-DC conversion efficiency represent only the performance of the passive RF energy harvesting chain comprising the antenna, impedance-matching network, and voltage-doubler rectifier, and therefore exclude the power consumed by the control circuitry. To further improve transparency, we have clarified that, in a fully autonomous battery-free implementation, the control circuitry would also require harvested energy or an alternative passive or duty-cycled switching strategy. Under such conditions, the net power available to the sensing system would be lower than the values reported in the present work. A corresponding discussion has also been added to the **Limitations** section (Section 4.6.2).

**Revision made**

- Section 2.1.1 was revised to clarify the experimental power supply arrangement.
- Section 4.6.2 was expanded to discuss the implications of powering the control circuitry in a fully autonomous battery-free implementation.

**Comment 3:** *Standardize the input power units in Table 8 for ease of comparison.*

**Response:** We thank the reviewer for this helpful recommendation. All input power values presented in Table 8 have been standardized to a single unit (µW) wherever possible. This revision improves consistency and facilitates direct comparison among the published studies. In addition, the manuscript was carefully reviewed to ensure consistent use of power units throughout.

**Revision made**

- Input power values in Table 8 were standardized to µW wherever applicable.
- Power units throughout the manuscript were reviewed and standardized for consistency.

**Comment 4:** *If Input Power Density (IPD) is unavailable for most studies, this parameter is not required in Table 8.*

**Response:** We agree with the reviewer's observation. After reviewing the cited studies, we found that Input Power Density (IPD) is not consistently reported in the literature. Therefore, the IPD column has been removed from Table 8 to improve consistency and simplify comparison among the published studies. The revised table now includes only those parameters that are consistently reported across the literature.

**Revision made**

- The IPD column was removed from Table 8.
- Table 8 was revised accordingly.

**Additional Revisions**

In addition to addressing the reviewers' comments, we have made the following improvements:

i. The Declaration of AI-Assisted Technologies was revised to improve transparency regarding the use of AI-assisted technologies.
ii. Figures 1 and 2 were completely redrawn by the authors and verified to comply with the PLOS ONE figure preparation guidelines.
iii. The manuscript was carefully reviewed to improve consistency in terminology, units, formatting, and presentation.

**Summary of Major Revisions**

| **Manuscript Section** | **Revision** |
| --- | --- |
| Figures 1 and 2 | Completely redrawn by the authors; Gemini watermark removed |
| Declaration of AI-Assisted Technologies | Revised for improved transparency |
| Section 2.1.1 | Clarified that the RF switch and microcontroller were externally powered during experimental characterization |
| Section 4.6.2 | Added discussion of control-circuit power consumption in future autonomous battery-free implementations |
| Table 8 | Standardized input power values to µW wherever applicable |
| Table 8 | Removed the IPD column |

We sincerely thank the Academic Editor and both reviewers for their careful evaluation and constructive comments. Their valuable suggestions have significantly improved the scientific quality, clarity, and transparency of our manuscript. We respectfully submit the revised manuscript for your consideration and hope that it is now suitable for publication in *PLOS ONE*.

Sincerely Yours,

Dr. Md. Firoz Ahmed (Corresponding Author)

On behalf of all authors

Department of Information and Communication Engineering

University of Rajshahi, Rajshahi-6205, Bangladesh

Email: [firozice01@ru.ac.bd](mailto:firozice01@ru.ac.bd)
