# Supplementary material for: Ambient RF energy harvesting using voltage doubler rectifier for battery-free agricultural sensor networks: Design, statistical analysis, and machine learning validation
Source: PLoS One. 2026 Sep 10;21(9):e0350236. doi: 10.1371/journal.pone.0350236 (PMC13561346; doi:10.1371/journal.pone.0350236)
Supplement: Peer review history

# Peer review: Decision Letter 0

**DOI:** 10.1371/journal.pone.0350236.r001

**Authors:** Sachin Kumar

23 Jun 2026

Ambient RF Energy Harvesting Using Voltage Doubler Rectifier for Battery-Free Agricultural Sensor Networks: Design, Statistical Analysis, and Machine Learning Validation

PLOS One

Dear Dr. Ahmed,

Thank you for submitting your manuscript to PLOS ONE. After careful consideration, we feel that it has merit but does not fully meet PLOS ONE’s publication criteria as it currently stands. Therefore, we invite you to submit a revised version of the manuscript that addresses the points raised during the review process.

Please submit your revised manuscript by Aug 22 2026 11:59PM. If you will need more time than this to complete your revisions, please reply to this message or contact the journal office at plosone@plos.org. When you're ready to submit your revision, log on to https://www.editorialmanager.com/pone/ and select the 'Submissions Needing Revision' folder to locate your manuscript file.

Please include the following items when submitting your revised manuscript:

- A letter that responds to each point raised by the academic editor and reviewer(s). You should upload this letter as a separate file labeled 'Response to Reviewers'.
- A marked-up copy of your manuscript that highlights changes made to the original version. You should upload this as a separate file labeled 'Revised Manuscript with Track Changes'.
- An unmarked version of your revised paper without tracked changes. You should upload this as a separate file labeled 'Manuscript'.

If you would like to make changes to your financial disclosure, please include your updated statement in your cover letter. Guidelines for resubmitting your figure files are available below the reviewer comments at the end of this letter.

If applicable, we recommend that you deposit your laboratory protocols in protocols.io to enhance the reproducibility of your results. Protocols.io assigns your protocol its own identifier (DOI) so that it can be cited independently in the future. For instructions see: https://journals.plos.org/plosone/s/submission-guidelines#loc-laboratory-protocols. Additionally, PLOS ONE offers an option for publishing peer-reviewed Lab Protocol articles, which describe protocols hosted on protocols.io. Read more information on sharing protocols at https://plos.org/protocols?utm_medium=editorial-email&utm_source=authorletters&utm_campaign=protocols.

As the corresponding author, your ORCID iD is verified in the submission system and will appear in the published article. PLOS supports the use of ORCID, and we encourage all coauthors to register for an ORCID iD and use it as well. Please encourage your coauthors to verify their ORCID iD within the submission system before final acceptance, as unverified ORCID iDs will not appear in the published article. *Only*  the individual author can complete the verification step; PLOS staff *cannot*  verify ORCID iDs on behalf of authors.

We look forward to receiving your revised manuscript.

Kind regards,

Sachin Kumar, Ph.D.

Academic Editor

PLOS One

Journal Requirements:

When submitting your revision, we need you to address these additional requirements.

1.Please ensure that your manuscript meets PLOS ONE's style requirements, including those for file naming. The PLOS ONE style templates can be found at

https://journals.plos.org/plosone/s/file?id=wjVg/PLOSOne_formatting_sample_main_body.pdf and

https://journals.plos.org/plosone/s/file?id=ba62/PLOSOne_formatting_sample_title_authors_affiliations.pdf

2. PLOS requires an ORCID iD for the corresponding author in Editorial Manager on papers submitted after December 6th, 2016. Please ensure that you have an ORCID iD and that it is validated in Editorial Manager. To do this, go to ‘Update my Information’ (in the upper left-hand corner of the main menu), and click on the Fetch/Validate link next to the ORCID field. This will take you to the ORCID site and allow you to create a new iD or authenticate a pre-existing iD in Editorial Manager.

3. Please note that PLOS One has specific guidelines on code sharing for submissions in which author-generated code underpins the findings in the manuscript. In these cases, we expect all author-generated code to be made available without restrictions upon publication of the work. Please review our guidelines at https://journals.plos.org/plosone/s/materials-and-software-sharing#loc-sharing-code and ensure that your code is shared in a way that follows best practice and facilitates reproducibility and reuse.

4. In the online submission form, you indicated that your data is available only on request from a third party. Please note that your Data Availability Statement is currently missing the name of the third party contact or institution / contact details for the third party, such as an email address or a link to where data requests can be made. Please update your statement with the missing information.

5. Please ensure that you refer to Figure 3 in your text as, if accepted, production will need this reference to link the reader to the figure.

6. If the reviewer comments include a recommendation to cite specific previously published works, please review and evaluate these publications to determine whether they are relevant and should be cited. There is no requirement to cite these works unless the editor has indicated otherwise.

Additional Editor Comments:

The reviewers have shown favorable interest in the submitted manuscript. However, substantial revisions are required to address the concerns and recommendations raised during the review process. The authors are therefore requested to thoroughly revise the manuscript in accordance with the reviewers’ comments and resubmit it for further evaluation.

[Note: HTML markup is below. Please do not edit.]

Reviewer's Responses to Questions

**Comments to the Author**

1. Is the manuscript technically sound, and do the data support the conclusions?

Reviewer #1: Yes

Reviewer #2: No

**********

2. Has the statistical analysis been performed appropriately and rigorously?

Reviewer #1: Yes

Reviewer #2: No

**********

3. Have the authors made all data underlying the findings in their manuscript fully available?

The PLOS Data policy

Reviewer #1: Yes

Reviewer #2: Yes

**********

4. Is the manuscript presented in an intelligible fashion and written in standard English?

Reviewer #1: Yes

Reviewer #2: Yes

**********

Reviewer #1: The authors presented their work on "Ambient RF Energy Harvesting Using Voltage Doubler Rectifier for Battery-Free Agricultural Sensor Networks: Design, Statistical Analysis, and Machine Learning Validation," which appears to be intriguing and significant. The manuscript is well written, and it has covered all the aspects, right from explaining the theoretical insights to its experimental validation from a conceptual perspective.

However, I believe the manuscript needs a few corrections:

a. The comparison table needs to include some recent state-of-the-art literature in chronological order (mention the year in the corresponding references).

b. Highlight the pros and cons of the proposed design in bulleted form over the ones reported earlier.

c. Do include additional insights regarding its novelty and application usage, which can benefit the upcoming readers in this particular field of interest.

So, these are the corrections that the authors need to make. Otherwise, the rest of the paper looks good to me.

Reviewer #2: The authors present a diode based multi-band ambient RF energy harvester targeting smart agricultural applications. The novelty here being the wide bandwidth being supported by the system (roughly 0.5GHz-2.1GHz) which covers AM, FM and cellular, relevant to the targeted rural to semi-urban, agricultural deployment conditions. Although an interesting concept, the authors do not provide sufficient explanation on how their novel approach is implemented and the results presented are vague, making comparisons across state-of the art unconvincing. Insufficient citations also make it hard for readers to verify the claims being presented by the authors.

I have the following comments regarding changes/additions that should be made to the manuscript before publication.

1. Please provide clear citations on the typical ambient RF power densities figure presented in the introduction.

2. In the introduction, the authors make the point that existing studies perform measurements/tests in controlled laboratory environments as a critical research gap, however in their own research findings, the results published are also performed under laboratory conditions. Either this research gap should be removed from the introduction, or authors should clarify why they were unable to provide real world measurement results which could fill the aforementioned research gap.

3. The authors indicate the multiband (0.5GHz to 2.1GHz) operation as a key functionality of the proposed system, with clear explanation on how its achieved on the antenna side (multiplexer), but none for the Impedance Matching Network. It is stated that “..network consists of an L-section (inductor-capacitor) optimized for each sub-band, achieving a VSWR of <2 across all target frequencies.” Considering that reactance variation from even just slight frequency shifts can cause significant mismatch, more elaboration is required on how the implemented L-matching network is able to cope with such a wide operating frequency. Additional clarification is also needed on whether the multi-band operation is tuned for a few key frequencies eg. 558MHz, 1089MHz, etc. OR for a wide-band approach where matching (VSWR<2) is achieved across the whole 0.5GHz to 2.1GHz spectrum.

4. Again, please provide clear citations on where the figures for typical RF Power densities in ambient conditions are obtained (Section 2.2).

5. Related to point 3, in Section 4, only one result is presented (mean output Voltage), despite the system being presented as a multiband. Please clarify how this presented mean is being calculated. A separate table/graph should also be included to present the results (VOC etc.) at/across all measured frequency points.

6. Provide citations for the <10µW average power draw of duty-cycled sensors figure stated in Section 4.2.

7. Fix the formatting for Table 4 and 6 (missing outlines).

8. Define the “abbreviation” ANOVA on first use.

9. Additional justification/explanation should be given at the start of Section 5 to highlight the significance of the Diode Performance analysis. Its current inclusion in the manuscript seems slightly out of place.

10. The parameters in the comparison table is lackluster and does not provide the readers with sufficient information to make informed judgement of the performance variation across compared works. At minimum, input power, rectifier type (on-chip, pcb etc.), should be stated. If possible, select other multi-band works to compare results. A few relevant multi-band (tri-band) works are listed below:

• N. J. Ubos, M. R. Zablan, K. D. Acas, O. J. Gerasta, G. F. Palencia, N. Mapula, K. Maglinte, R. Sabarillo, and J. L. Balassa, ‘‘RF-to-DC conversion circuit with configurable impedance match ing circuit for RF energy harvesting in 65nm CMOS technology,’’ in Proc. TENCON IEEE Region Conf. (TENCON), Singapore, Dec. 2024, pp. 1314–1318.

• J. K. Yong, W. X. Lian, H. Ramiah, K. K. P. Churchill, G. Chong, N. S. Lai, Y. Chen, P.-I. Mak, and R. P. Martins, ‘‘A fully integrated CMOS tri-band ambient RF energy harvesting system for IoT devices,’’ IEEE Trans. Circuits Syst. I, Reg. Papers, vol. 70, no. 12, pp. 4705–4718, Dec. 2023.

11. Again, please show the results at each/fixed frequencies instead of the mean when presenting for comparisons.

12. Without comparing input conditions, the statement, “The proposed system delivers the highest broadband operational frequency coverage (558 kHz–2.1 GHz), the highest open circuit voltage (4.07 V), and the highest loaded output voltage (2.11 V) among the compared designs.”, does not hold water and should be expanded on.

13. Measurement results of the lower input power conditions (3db-10db lower) should be presented to back the claim of the 2Vloaded being sufficient to overcome the “Foliage Attenuation” stated in Section 4.6.

14. “Geographic Relevance” in Section 4.6 is not a relevant claim as the measurements were still conducted in a laboratory environment. Either remove or reword this statement.

**********

what does this mean?). If published, this will include your full peer review and any attached files.

If you choose “no”, your identity will remain anonymous but your review may still be made public.

**Do you want your identity to be public for this peer review?**  For information about this choice, including consent withdrawal, please see our Privacy Policy

Reviewer #1: No

Reviewer #2: No

**********

[NOTE: If reviewer comments were submitted as an attachment file, they will be attached to this email and accessible via the submission site. Please log into your account, locate the manuscript record, and check for the action link "View Attachments". If this link does not appear, there are no attachment files.]

To ensure your figures meet our technical requirements, please review our figure guidelines: https://journals.plos.org/plosone/s/figures

You may also use PLOS’s free figure tool, NAAS, to help you prepare publication quality figures: https://journals.plos.org/plosone/s/figures#loc-tools-for-figure-preparation.

NAAS will assess whether your figures meet our technical requirements by comparing each figure against our figure specifications.

# Author comment: Author response to Decision Letter 1

**DOI:** 10.1371/journal.pone.0350236.r002

14 Jul 2026

Response to Reviewers

Manuscript ID: PONE-D-26-23129

Title: Ambient RF Energy Harvesting Using Voltage Doubler Rectifier for Battery-Free Agricultural Sensor Networks: Design, Statistical Analysis, and Machine Learning Validation

Authors: Md. Atik Hasan Nishat, Prithwiraj Biswas Pallab, Nowrin Jannat, Saleha Nasrin Mishu, Md Fahad Ullah Utsho, Md. Bipul Islam, Riaz Uddin Mondal, Md. Firoz Ahmed, M. Hasnat Kabir

Dear Editor-in-Chief,

We sincerely thank the Academic Editor and both reviewers for their thorough and constructive evaluation of our manuscript. Their insightful comments and recommendations have substantially improved the quality, clarity, and rigor of our work. Below, we provide a detailed, point-by-point response to each comment raised during the review process. All changes made in the revised manuscript have been highlighted in red.

Response to Journal Requirements

1. PLOS ONE style requirements:

Response: We have carefully reviewed and formatted the manuscript according to the PLOS ONE style templates, ensuring compliance with all file naming and formatting guidelines.

2. ORCID iD validation:

Response: The corresponding author (Dr. Md. Firoz Ahmed) has an ORCID iD, and it has been successfully validated in the Editorial Manager system.

3. Code sharing guidelines:

Response: We acknowledge PLOS ONE’s guidelines on code sharing. In this study, the machine learning and statistical analyses were performed using standard, widely available open-source libraries (e.g., scikit-learn, XGBoost, SHAP, and standard scientific computing tools in Python/R). No custom, author-generated proprietary code underpins the core findings; therefore, specific code deposition is not applicable. However, the experimental data and analytical workflows are available upon request as detailed in the Data Availability Statement.

4. Data Availability Statement update:

Response: We have updated the Data Availability Statement to include the specific contact details for data requests. It now reads: “The experimental data supporting the findings of this study are available upon reasonable request. For institutional oversight and long-term data stewardship, data requests should be directed to the Department of Information and Communication Engineering, University of Rajshahi, Rajshahi-6205, Bangladesh (Email: ice@ru.ac.bd), which serves as the primary institutional non-author point of contact for managing data requests. Requests may also be directed to the corresponding author, Dr. Md. Firoz Ahmed (Email: firozice01@ru.ac.bd). The experimental measurement data contain no personally identifiable information or other ethical restrictions and are available for verification and reproducibility purposes upon reasonable request.”

5. Reference to Figure 3:

Response: We have ensured that Figure 3 is explicitly referenced in the text (Section 4.1, paragraph 1) as required for production linking.

6. Review of recommended citations:

Response: We thank the editor for pointing this out. We have carefully reviewed the recommended publications and found them highly relevant to our work on multi-band RF energy harvesting. We have incorporated citations for the suggested multi-band/tri-band works by Ubos et al. and Yong et al. into the manuscript (now References [13] and [14]) and included them in our comparative analysis.

Response to Reviewer #1

We thank the reviewer for their positive assessment of our work and for recognizing the significance and comprehensive nature of our study. We have addressed the suggested corrections as follows:

Comment a: The comparison table needs to include some recent state-of-the-art literature in chronological order (mention the year in the corresponding references).

Response: We agree with this suggestion. Table 8 has been updated to include recent state-of-the-art literature (spanning from 2022 to 2026). A dedicated “Year” column has been added, and the references are now arranged in chronological order to provide a clear timeline of technological advancements in this field.

Comment b: Highlight the pros and cons of the proposed design in bulleted form over the ones reported earlier.

Response: We have added a new subsection (Section 4.6) that explicitly outlines the strengths and limitations of our proposed design in a clear, bulleted format. Section 4.6.1 details the pros (e.g., broad frequency coverage, low-cost hardware, comprehensive statistical validation), while Section 4.6.2 addresses the cons (e.g., lower conversion efficiency compared to CMOS, larger hardware footprint).

Comment c: Do include additional insights regarding its novelty and application usage, which can benefit the upcoming readers in this particular field of interest.

Response: We have added Section 4.8, "Potential Impact on Agricultural IoT Deployment," to highlight the practical novelty and application usage of the proposed system. This section discusses how the harvester can support self-powered sensor nodes for smallholder farming environments in South Asia, reducing dependence on battery replacement and enabling scalable precision agriculture.

Response to Reviewer #2

We thank the reviewer for their critical and detailed evaluation, which has helped us significantly clarify our methodology, address ambiguities, and strengthen the manuscript's technical rigor.

Comment 1: Please provide clear citations on the typical ambient RF power densities figure presented in the introduction.

Response: We have added appropriate citations [5–8] in the Introduction to support the stated typical ambient RF power density ranges (0.1–10 µW/cm² in rural environments and higher in semi-urban areas).

Comment 2: In the introduction, the authors make the point that existing studies perform measurements/tests in controlled laboratory environments as a critical research gap, however in their own research findings, the results published are also performed under laboratory conditions. Either this research gap should be removed from the introduction, or authors should clarify why they were unable to provide real world measurement results which could fill the aforementioned research gap.

Response: We appreciate this valid observation. We have clarified this point in both the Introduction and Section 4.1. We explained that while long-term outdoor field measurements are difficult to control and isolate due to extreme environmental variability, our laboratory setup was specifically designed to emulate realistic ambient RF variability using multi-frequency excitation and controlled time-varying power levels (frequency sweeping and pseudo-random amplitude modulation). This allows for repeatable, statistically rigorous experimentation that approximates real-world fluctuations, serving as a necessary foundational step before long-term field deployment.

Comment 3: The authors indicate the multiband (0.5GHz to 2.1GHz) operation as a key functionality of the proposed system, with clear explanation on how its achieved on the antenna side (multiplexer), but none for the Impedance Matching Network. It is stated that “. network consists of an L-section (inductor-capacitor) optimized for each sub-band, achieving a VSWR of <2 across all target frequencies.” Considering that reactance variation from even just slight frequency shifts can cause significant mismatch, more elaboration is required on how the implemented L-matching network is able to cope with such a wide operating frequency. Additional clarification is also needed on whether the multi-band operation is tuned for a few key frequencies eg. 558MHz, 1089MHz, etc. OR for a wide-band approach where matching (VSWR<2) is achieved across the whole 0.5GHz to 2.1GHz spectrum.

Response: We have significantly expanded Section 2.1.1 to clarify this. We explicitly state that instead of using a single broadband matching network (which suffers from severe reactance variations over ultra-wide ranges), we implemented three dedicated, switchable L-section impedance-matching networks. Each network is independently optimized for a specific sub-band (AM, FM, and Cellular). An RF switch, controlled by a microcontroller, selects the appropriate branch based on signal strength. Thus, the system achieves VSWR < 2 across the entire spectrum by tuning discrete narrow-to-mid bands sequentially, rather than attempting a single wide-band match.

Comment 4: Again, please provide clear citations on where the figures for typical RF Power densities in ambient conditions are obtained (Section 2.2).

Response: We have added citations [5–8] in Section 2.2 to justify the selected RF power density range (0.1–5 µW/cm²) based on previous measurement studies in comparable South Asian environments.

Comment 5: Related to point 3, in Section 4, only one result is presented (mean output Voltage), despite the system being presented as a multiband. Please clarify how this presented mean is being calculated. A separate table/graph should also be included to present the results (VOC etc.) at/across all measured frequency points.

Response: We have added Section 4.2.1, “Frequency-Dependent Performance Analysis,” to explain that the overall mean is calculated by equally weighting the means of representative frequencies across the bands. Furthermore, we have added Table 3a, which explicitly presents the loaded output voltage and harvested power at each specific measured frequency point (639 kHz, 98 MHz, 900 MHz, 1800 MHz, and 2100 MHz).

Comment 6: Provide citations for the <10µW average power draw of duty-cycled sensors figure stated in Section 4.2.

Response: We have added Reference [9] in Section 4.2 to support the claim regarding the average power requirements of aggressively duty-cycled environmental sensing systems.

Comment 7: Fix the formatting for Table 4 and 6 (missing outlines).

Response: The formatting for Table 4 and Table 6 has been corrected, and all table outlines/borders are now properly included in the revised manuscript.

Comment 8: Define the “abbreviation” ANOVA on first use.

Response: We have defined "Analysis of Variance (ANOVA)" upon its first use in both the Abstract and the Introduction.

Comment 9: Additional justification/explanation should be given at the start of Section 5 [now Section 4.3] to highlight the significance of the Diode Performance analysis. Its current inclusion in the manuscript seems slightly out of place.

Response: We have rewritten the introductory paragraph of Section 4.3 to clearly justify the importance of diode selection. We now explain that under ultra-low ambient RF conditions, rectification losses are the primary bottleneck, and the diode's forward voltage directly dictates the minimum sensitivity threshold of the harvester. This contextualizes why a rigorous statistical comparison of diodes is critical for agricultural deployments in remote, weak-signal areas.

Comment 10: The parameters in the comparison table is lackluster and does not provide the readers with sufficient information to make informed judgement of the performance variation across compared works. At minimum, input power, rectifier type (on-chip, pcb etc.), should be stated. If possible, select other multi-band works to compare results. A few relevant multi-band (tri-band) works are listed below:

• N. J. Ubos, M. R. Zablan, K. D. Acas, O. J. Gerasta, G. F. Palencia, N. Mapula, K. Maglinte, R. Sabarillo, and J. L. Balassa, ‘‘RF-to-DC conversion circuit with configurable impedance match ing circuit for RF energy harvesting in 65nm CMOS technology,’’ in Proc. TENCON IEEE Region Conf. (TENCON), Singapore, Dec. 2024, pp. 1314–1318.

• J. K. Yong, W. X. Lian, H. Ramiah, K. K. P. Churchill, G. Chong, N. S. Lai, Y. Chen, P.-I. Mak, and R. P. Martins, ‘‘A fully integrated CMOS tri-band ambient RF energy harvesting system for IoT devices,’’ IEEE Trans. Circuits Syst. I, Reg. Papers, vol. 70, no. 12, pp. 4705–4718, Dec. 2023.

Response: We have completely overhauled Table 8. We added columns for Input Power Density (IPD), Input Power (IP), Rectifier Type (RT - specifying PCB discrete vs. CMOS IC), and Band Coverage (BC). We have also included the highly relevant multi-band/tri-band works suggested by the reviewer (Yong et al. [13] and Ubos et al. [14]) to provide a much more informed and comprehensive comparison.

Comment 11: Again, please show the results at each/fixed frequencies instead of mean when presenting for comparisons.

Response: As addressed in Comment 5, we have added Table 3a to show the exact results at each fixed frequency. In the revised comparison discussion (Section 4.5), we now explicitly caution readers against direct numerical comparisons of our overall mean with other works' single-frequency results, and instead direct readers to Table 3a for our frequency-specific performance metrics.

Comment 12: Without comparing input conditions, the statement, “The proposed system delivers the highest broadband operational frequency coverage (558 kHz–2.1 GHz), the highest open circuit voltage (4.07 V), and the highest loaded output voltage (2.11 V) among the compared designs.”, does not hold water and should be expanded on.

Response: We completely agree. We have revised this statement in Section 4.5. We now explicitly state that because the compared studies differ vastly in input power, antenna characteristics, and measurement methodologies, direct numerical comparisons of output voltage must be interpreted with caution. We have reframed the contribution to emphasize that our main advantage lies in the combination of ultra-wide frequency coverage, low-cost discrete implementation, and a rigorous statistical/ML validation framework, rather than claiming absolute superiority in voltage output under mismatched input conditions.

Comment 13: Measurement results of the lower input power conditions (3db-10db lower) should be presented to back the claim of the 2V loaded being sufficient to overcome the “Foliage Attenuation” stated in Section 4.6 [now 4.7.2].

Response: We have added a new subsection, Section 4.7.2 “Foliage-Induced Signal Attenuation,” which includes Table 9. This table presents the measured loaded output voltage and harvested power under simulated foliage attenuation conditions (0 dB baseline, 3 dB reduction, and 10 dB reduction), proving that the harvester remains operational and above the minimum supply thresholds for ultra-low-power sensors even under severe attenuation.

Comment 14: “Geographic Relevance” in Section 4.6 is not a relevant claim as the measurements were still conducted in a laboratory environment. Either remove or reword this statement.

Response: We have removed the term “Geographic Relevance.” The section has been reworded and renamed to “Applicability to Rural and Semi-Urban Agricultural Regions” (Section 4.7.3). We explicitly clarify that the laboratory evaluation was designed using RF power densities representative of literature-reported conditions in these regions, providing a laboratory-based indication of expected behavior rather than claiming direct geographic field validation.

Response to Specific Editorial Team Comments

Comment 1: Please ensure that you refer to Figure 1 in your text as, if accepted, production will need this reference to link the reader to the figure.

Author Response: We appreciate the editorial team’s observation and have addressed this issue by incorporating an explicit reference to Figure 1 in the revised manuscript.

Revision Made: A reference to Figure 1 has been added in Section 2.1 (System Architecture Overview). The following sentence has been included:

“Figure 1 illustrates the block diagram of the proposed RF energy harvesting system, depicting the signal flow from RF energy capture through impedance matching, rectification, and energy storage stages. The architecture demonstrates the conversion pathway of ambient RF signals into usable DC power for supplying low-power agricultural sensor nodes.”

Location in Revised Manuscript: Section 2.1, Paragraph 2.

Evidence of Change: The citation of Figure 1 has been incorporated into the main text before the figure placement, enabling appropriate cross-referencing and production linking.

Comment 2: Please ensure that Table 7 is referenced in the main text, as this reference is required during production to enable linking between the text

Attachment Submitted filename: Response to Reviewers.docx

# Peer review: Decision Letter 1

**DOI:** 10.1371/journal.pone.0350236.r003

**Authors:** Sachin Kumar

27 Jul 2026

Dear Dr. Ahmed,

Thank you for submitting your manuscript to PLOS ONE. After careful consideration, we feel that it has merit but does not fully meet PLOS ONE’s publication criteria as it currently stands. Therefore, we invite you to submit a revised version of the manuscript that addresses the points raised during the review process.

Please submit your revised manuscript by Aug 26 2026 11:59PM. If you will need more time than this to complete your revisions, please reply to this message or contact the journal office at plosone@plos.org. When you're ready to submit your revision, log on to https://www.editorialmanager.com/pone/ and select the 'Submissions Needing Revision' folder to locate your manuscript file.

- A letter that responds to each point raised by the academic editor and reviewer(s). You should upload this letter as a separate file labeled 'Response to Reviewers'.
- A marked-up copy of your manuscript that highlights changes made to the original version. You should upload this as a separate file labeled 'Revised Manuscript with Track Changes'.
- An unmarked version of your revised paper without tracked changes. You should upload this as a separate file labeled 'Manuscript'.

If you would like to make changes to your financial disclosure, please include your updated statement in your cover letter. Guidelines for resubmitting your figure files are available below the reviewer comments at the end of this letter.

If applicable, we recommend that you deposit your laboratory protocols in protocols.io to enhance the reproducibility of your results. Protocols.io assigns your protocol its own identifier (DOI) so that it can be cited independently in the future. For instructions see: https://journals.plos.org/plosone/s/submission-guidelines#loc-laboratory-protocols. Additionally, PLOS ONE offers an option for publishing peer-reviewed Lab Protocol articles, which describe protocols hosted on protocols.io. Read more information on sharing protocols at https://plos.org/protocols?utm_medium=editorial-email&utm_source=authorletters&utm_campaign=protocols.

As the corresponding author, your ORCID iD is verified in the submission system and will appear in the published article. PLOS supports the use of ORCID, and we encourage all coauthors to register for an ORCID iD and use it as well. Please encourage your coauthors to verify their ORCID iD within the submission system before final acceptance, as unverified ORCID iDs will not appear in the published article. *Only*  the individual author can complete the verification step; PLOS staff *cannot*  verify ORCID iDs on behalf of authors.

We look forward to receiving your revised manuscript.

Kind regards,

Sachin Kumar, Ph.D.

Academic Editor

PLOS One

Journal Requirements:

If the reviewer comments include a recommendation to cite specific previously published works, please review and evaluate these publications to determine whether they are relevant and should be cited. There is no requirement to cite these works unless the editor has indicated otherwise.

Please review your reference list to ensure that it is complete and correct. If you have cited papers that have been retracted, please include the rationale for doing so in the manuscript text, or remove these references and replace them with relevant current references. Any changes to the reference list should be mentioned in the rebuttal letter that accompanies your revised manuscript. If you need to cite a retracted article, indicate the article’s retracted status in the References list and also include a citation and full reference for the retraction notice.

Additional Editor Comments:

The manuscript has been significantly improved. However, the reviewers have raised a few additional concerns that require further clarification. Please address all the reviewer comments comprehensively and revise the manuscript accordingly to strengthen its quality before resubmission.

[Note: HTML markup is below. Please do not edit.]

Reviewers' comments:

Reviewer's Responses to Questions

**Comments to the Author**

Reviewer #1: All comments have been addressed

Reviewer #2: (No Response)

**********

2. Is the manuscript technically sound, and do the data support the conclusions?

Reviewer #1: Yes

Reviewer #2: Yes

**********

3. Has the statistical analysis been performed appropriately and rigorously?

Reviewer #1: N/A

Reviewer #2: Yes

**********

4. Have the authors made all data underlying the findings in their manuscript fully available?

The PLOS Data policy

Reviewer #1: Yes

Reviewer #2: Yes

**********

5. Is the manuscript presented in an intelligible fashion and written in standard English?

Reviewer #1: Yes

Reviewer #2: Yes

**********

Reviewer #1: The authors presented their work on "Ambient RF Energy Harvesting Using Voltage Doubler Rectifier for Battery-Free Agricultural Sensor Networks: Design, Statistical Analysis, and Machine Learning Validation," which seems to be quite interesting and significant. The manuscript is well written and has covered all the aspects, right from explaining the theoretical insights to experimental validation from a conceptual point of view.

Moreover, what I observed was that they had addressed all the concerns raised by the reviewers in the early stage of the review process.

Now, the paper looks good to me. So, good luck to the authors.

Reviewer #2: The authors present a diode based multi-band ambient RF energy harvester targeting smart agricultural applications. The novelty here being the wide bandwidth being supported by the system (roughly 0.5GHz-2.1GHz) which covers AM, FM and cellular, relevant to the targeted rural to semi-urban, agricultural deployment conditions. The authors have sufficiently addressed the previous comments received but there are a few additional ones listed below, with the only major concern being the lack of disclosure on the use of generative AI (Google’s Gemini) for certain figures.

1. The Gemini water mark is present in the bottom right corner of Figure 1 & Figure 2, indicating the figure was AI generated/altered. As no disclosure of this was made by the authors, either remove or replace the affected images. Authors merely stated “The authors used ChatGPT solely for language editing and grammatical refinement of the manuscript. All aspects of the study, including the research design, data collection, experimental implementation, statistical analysis, interpretation of results, and scientific conclusions, were performed independently by the authors.”

2. Does the reported efficiency/output-power take into account the power consumption of the control circuit of the frequency selection circuits (RF switch & microcontroller). I assume they are powered by the output power generated by the rectifier itself? If so please do make it clear in section 2.1.1, where the control circuit is first presented.

3. Standardize the units for input power in Table 8 (convert all to watts OR dBm) for ease of comparison. Try to keep consistent units for all presented parameters throughout the manuscript.

4. If all compared works do not present available IPD, the parameter is not required to be presented in the comparison table 8.

**********

what does this mean?). If published, this will include your full peer review and any attached files.

If you choose “no”, your identity will remain anonymous but your review may still be made public.

**Do you want your identity to be public for this peer review?**  For information about this choice, including consent withdrawal, please see our Privacy Policy

Reviewer #1: No

Reviewer #2: No

**********

[NOTE: If reviewer comments were submitted as an attachment file, they will be attached to this email and accessible via the submission site. Please log into your account, locate the manuscript record, and check for the action link "View Attachments". If this link does not appear, there are no attachment files.]

To ensure your figures meet our technical requirements, please review our figure guidelines: https://journals.plos.org/plosone/s/figures

You may also use PLOS’s free figure tool, NAAS, to help you prepare publication quality figures: https://journals.plos.org/plosone/s/figures#loc-tools-for-figure-preparation.

NAAS will assess whether your figures meet our technical requirements by comparing each figure against our figure specifications.

# Author comment: Author response to Decision Letter 2

**DOI:** 10.1371/journal.pone.0350236.r004

31 Jul 2026

Response to Reviewers

Manuscript ID: PONE-D-26-23129R1

Title: Ambient RF Energy Harvesting Using Voltage Doubler Rectifier for Battery-Free Agricultural Sensor Networks: Design, Statistical Analysis, and Machine Learning Validation

Journal: PLOS ONE

Dear Editor-in-Chief,

We sincerely thank you and the reviewers for your careful evaluation of our manuscript and for your constructive comments and valuable suggestions. We greatly appreciate the time and effort devoted to reviewing our work. The comments have helped us improve the clarity, transparency, and overall quality of the manuscript. We have carefully considered all comments and revised the manuscript accordingly. A detailed point-by-point response to each comment has been prepared and submitted as a separate Response to Reviewers document. For ease of evaluation, all revisions in the marked-up manuscript have been highlighted in red.

Reviewer #1

Comment: The reviewer considered the manuscript interesting and significant, noted that it is well written, and indicated that all previous concerns had been satisfactorily addressed.

Response: We sincerely thank Reviewer #1 for the positive assessment and encouraging comments. We greatly appreciate the reviewer's recognition that the concerns raised during the previous review rounds have been satisfactorily addressed. We are grateful for the constructive feedback provided throughout the review process, which has substantially improved the quality of our manuscript.

Reviewer #2

Comment 1: The Gemini watermark is present in Figures 1 and 2, indicating that the figures were AI generated or altered. Please remove or replace the affected figures and provide an appropriate disclosure regarding AI-assisted technologies.

Response: We thank the reviewer for identifying this important issue. Figures 1 and 2 have been completely redrawn by the authors using Microsoft Word 2016. The previous figures containing the Gemini watermark have been removed entirely and replaced with original figures prepared by the authors. Consequently, no AI-generated or AI-modified figures are included in the revised manuscript. The Declaration of AI-Assisted Technologies has also been revised and now states:

The authors used ChatGPT solely for language editing and grammatical refinement of the manuscript. All aspects of the study, including the research design, experimental implementation, data collection, statistical analysis, interpretation of the results, preparation of figures, and scientific conclusions, were performed independently by the authors. No generative AI image tools were used in the preparation of the figures included in this manuscript.

We believe these revisions fully address the reviewer's concern regarding transparency and compliance with the journal's policy on AI-assisted technologies.

Revision made

� Figures 1 and 2 were completely redrawn by the authors.

� The Gemini watermarks were removed.

� The Declaration of AI-Assisted Technologies was revised to improve transparency regarding the use of AI-assisted technologies.

Comment 2: Please clarify whether the reported output power and RF-to-DC conversion efficiency include the power consumption of the RF switch and microcontroller used for frequency-band selection.

Response: We appreciate this valuable suggestion. Section 2.1.1 has been revised to explicitly clarify that the RF switch and microcontroller were powered by an external regulated laboratory power supply during experimental characterization. Consequently, the reported open-circuit voltage, loaded output voltage, harvested power, and RF-to-DC conversion efficiency represent only the performance of the passive RF energy harvesting chain comprising the antenna, impedance-matching network, and voltage-doubler rectifier, and therefore exclude the power consumed by the control circuitry. To further improve transparency, we have clarified that, in a fully autonomous battery-free implementation, the control circuitry would also require harvested energy or an alternative passive or duty-cycled switching strategy. Under such conditions, the net power available to the sensing system would be lower than the values reported in the present work. A corresponding discussion has also been added to the Limitations section (Section 4.6.2).

Revision made

� Section 2.1.1 was revised to clarify the experimental power supply arrangement.

� Section 4.6.2 was expanded to discuss the implications of powering the control circuitry in a fully autonomous battery-free implementation.

Comment 3: Standardize the input power units in Table 8 for ease of comparison.

Response: We thank the reviewer for this helpful recommendation. All input power values presented in Table 8 have been standardized to a single unit (µW) wherever possible. This revision improves consistency and facilitates direct comparison among the published studies. In addition, the manuscript was carefully reviewed to ensure consistent use of power units throughout.

Revision made

� Input power values in Table 8 were standardized to µW wherever applicable.

� Power units throughout the manuscript were reviewed and standardized for consistency.

Comment 4: If Input Power Density (IPD) is unavailable for most studies, this parameter is not required in Table 8.

Response: We agree with the reviewer's observation. After reviewing the cited studies, we found that Input Power Density (IPD) is not consistently reported in the literature. Therefore, the IPD column has been removed from Table 8 to improve consistency and simplify comparison among the published studies. The revised table now includes only those parameters that are consistently reported across the literature.

Revision made

� The IPD column was removed from Table 8.

� Table 8 was revised accordingly.

Additional Revisions

In addition to addressing the reviewers' comments, we have made the following improvements:

i. The Declaration of AI-Assisted Technologies was revised to improve transparency regarding the use of AI-assisted technologies.

ii. Figures 1 and 2 were completely redrawn by the authors and verified to comply with the PLOS ONE figure preparation guidelines.

iii. The manuscript was carefully reviewed to improve consistency in terminology, units, formatting, and presentation.

Summary of Major Revisions

Manuscript Section Revision

Figures 1 and 2 Completely redrawn by the authors; Gemini watermark removed

Declaration of AI-Assisted Technologies Revised for improved transparency

Section 2.1.1 Clarified that the RF switch and microcontroller were externally powered during experimental characterization

Section 4.6.2 Added discussion of control-circuit power consumption in future autonomous battery-free implementations

Table 8 Standardized input power values to µW wherever applicable

Table 8 Removed the IPD column

We sincerely thank the Academic Editor and both reviewers for their careful evaluation and constructive comments. Their valuable suggestions have significantly improved the scientific quality, clarity, and transparency of our manuscript. We respectfully submit the revised manuscript for your consideration and hope that it is now suitable for publication in PLOS ONE.

Sincerely Yours,

Dr. Md. Firoz Ahmed (Corresponding Author)

On behalf of all authors

Department of Information and Communication Engineering

University of Rajshahi, Rajshahi-6205, Bangladesh

Email: firozice01@ru.ac.bd

Attachment Submitted filename: Response_to_Reviewers_auresp_2.docx

# Peer review: Decision Letter 2

**DOI:** 10.1371/journal.pone.0350236.r005

**Authors:** Sachin Kumar

23 Aug 2026

Ambient RF Energy Harvesting Using Voltage Doubler Rectifier for Battery-Free Agricultural Sensor Networks: Design, Statistical Analysis, and Machine Learning Validation

PONE-D-26-23129R2

Dear Dr. Ahmed,

We’re pleased to inform you that your manuscript has been judged scientifically suitable for publication and will be formally accepted for publication once it meets all outstanding technical requirements.

Within one week, you’ll receive an e-mail detailing the required amendments. When these have been addressed, you’ll receive a formal acceptance letter and your manuscript will be scheduled for publication.

An invoice will be generated when your article is formally accepted. Please note, if your institution has a publishing partnership with PLOS and your article meets the relevant criteria, all or part of your publication costs will be covered. Please make sure your user information is up-to-date by logging into Editorial Manager at Editorial Manager® and clicking the ‘Update My Information' link at the top of the page. For questions related to billing, please contact billing support.

If your institution or institutions have a press office, please notify them about your upcoming paper to help maximize its impact. If they’ll be preparing press materials, please inform our press team as soon as possible -- no later than 48 hours after receiving the formal acceptance. Your manuscript will remain under strict press embargo until 2 pm Eastern Time on the date of publication. For more information, please contact onepress@plos.org.

Kind regards,

Sachin Kumar, Ph.D.

Academic Editor

PLOS One

Additional Editor Comments (optional):

The authors have carefully addressed all the comments raised during the review process, and the revised version satisfactorily resolves the reviewers' concerns. The reviewers are satisfied with the revisions provided by the authors, and the manuscript is now suitable for further consideration.

Reviewers' comments:

Reviewer's Responses to Questions

**Comments to the Author**

Reviewer #1: All comments have been addressed

Reviewer #2: All comments have been addressed

**********

2. Is the manuscript technically sound, and do the data support the conclusions?

Reviewer #1: Yes

Reviewer #2: Yes

**********

3. Has the statistical analysis been performed appropriately and rigorously?

Reviewer #1: N/A

Reviewer #2: Yes

**********

4. Have the authors made all data underlying the findings in their manuscript fully available?

The PLOS Data policy

Reviewer #1: Yes

Reviewer #2: Yes

**********

5. Is the manuscript presented in an intelligible fashion and written in standard English?

Reviewer #1: Yes

Reviewer #2: Yes

**********

Reviewer #1: The authors presented their work on "Ambient RF Energy Harvesting Using Voltage Doubler Rectifier for Battery-Free Agricultural Sensor Networks: Design, Statistical Analysis, and Machine Learning Validation," which seems to be quite interesting and significant. The manuscript is well written and has covered all the aspects, right from explaining the theoretical insights to its implementation from a conceptual point of view.

Moreover, what I observed was that they had addressed all the concerns raised by the reviewers in the early stage of the review process.

Now, the paper looks good to me. So, good luck to the authors.

Reviewer #2: (No Response)

**********

what does this mean?). If published, this will include your full peer review and any attached files.

If you choose “no”, your identity will remain anonymous but your review may still be made public.

**Do you want your identity to be public for this peer review?**  For information about this choice, including consent withdrawal, please see our Privacy Policy

Reviewer #1: No

Reviewer #2: No

**********

# Editor report: Acceptance letter

**DOI:** 10.1371/journal.pone.0350236.r006

**Authors:** Sachin Kumar

PONE-D-26-23129R2

PLOS One

Dear Dr. Ahmed,

I'm pleased to inform you that your manuscript has been deemed suitable for publication in PLOS One. Congratulations! Your manuscript is now being handed over to our production team.

At this stage, our production department will prepare your paper for publication. This includes ensuring the following:

* All references, tables, and figures are properly cited

* All relevant supporting information is included in the manuscript submission,

* There are no issues that prevent the paper from being properly typeset

You will receive further instructions from the production team, including instructions on how to review your proof when it is ready. Please keep in mind that we are working through a large volume of accepted articles, so please give us a few days to review your paper and let you know the next and final steps.

Lastly, if your institution or institutions have a press office, please let them know about your upcoming paper now to help maximize its impact. If they'll be preparing press materials, please inform our press team within the next 48 hours. Your manuscript will remain under strict press embargo until 2 pm Eastern Time on the date of publication. For more information, please contact onepress@plos.org.

You will receive an invoice from PLOS for your publication fee after your manuscript has reached the completed accept phase. If you receive an email requesting payment before acceptance or for any other service, this may be a phishing scheme. Learn how to identify phishing emails and protect your accounts at https://explore.plos.org/phishing.

If we can help with anything else, please email us at customercare@plos.org.

Thank you for submitting your work to PLOS One and supporting open access.

Kind regards,

PLOS One Editorial Office Staff

on behalf of

Dr. Sachin Kumar

Academic Editor

PLOS One
